# Insights from GWAS: emerging landscape of mechanisms underlying complex trait disease

**DOI:** 10.1186/1471-2164-16-S8-S4

**Published:** 2015-06-18

**Authors:** Lipika R Pal, Chen-Hsin Yu, Stephen M Mount, John Moult

**Affiliations:** 1 Institute for Bioscience and Biotechnology Research, University of Maryland, Rockville, MD, USA; 2 Molecular and Cellular Biology Program, University of Maryland, College Park, MD, USA; 3 Department of Cell Biology and Molecular Genetics, University of Maryland, College Park, MD, USA; 4 Center for Bioinformatics and Computational Biology, University of Maryland at College Park, College Park, MD, USA

**Keywords:** GWAS, SNPs, complex trait disease, missense, expression, splicing, functional annotation

## Abstract

**Background:**

There are now over 2000 loci in the human genome where genome wide association studies (GWAS) have found one or more SNPs to be associated with altered risk of a complex trait disease. At each of these loci, there must be some molecular level mechanism relevant to the disease. What are these mechanisms and how do they contribute to disease?

**Results:**

Here we consider the roles of three primary mechanism classes: changes that directly alter protein function (missense SNPs), changes that alter transcript abundance as a consequence of variants close-by in sequence, and changes that affect splicing. Missense SNPs are divided into those predicted to have a high impact on *in vivo *protein function, and those with a low impact. Splicing is divided into SNPs with a direct impact on splice sites, and those with a predicted effect on auxiliary splicing signals. The analysis was based on associations found for seven complex trait diseases in the classic Wellcome Trust Case Control Consortium (WTCCC1) GWA study and subsequent studies and meta-analyses, collected from the GWAS catalog. Linkage disequilibrium information was used to identify possible candidate SNPs for involvement in disease mechanism in each of the 356 loci associated with these seven diseases. With the parameters used, we find that 76% of loci have at least of these mechanisms. Overall, except for the low incidence of direct impact on splice sites, the mechanisms are found at similar frequencies, with changes in transcript abundance the most common. But the distribution of mechanisms over diseases varies markedly, as does the fraction of loci with assigned mechanisms. Many of the implicated proteins have previously been suggested as relevant, but the specific mechanism assignments are new. In addition, a number of new disease relevant proteins are proposed.

**Conclusions:**

The high fraction of GWAS loci with proposed mechanisms suggests that these classes of mechanism play a major role. Other mechanism types, such as variants affecting expression of genes remote in the DNA sequence, will contribute in other loci. Each of the identified putative mechanisms provides a hypothesis for further investigation.

## Background

There is now an explosion of new genome-scale data relating genetic variation within human populations to phenotype, and particularly to common disease. To date, most information has been obtained through genome wide association studies (GWAS) linking common single nucleotide variants (the single nucleotide polymorphisms, SNPs) to disease risk. The results from all these new data are tantalizing - on the one hand we now have extensive, reliable information on the associations between particular genetic variants and phenotypes. On the other, few of these associations provide any insight into the mechanisms linking genetic variation to phenotype. As a consequence, there are few immediate impacts on health in terms of improved therapies, reliable prognosis, or other benefits.

An important step in exploiting the data is to search for mechanisms that link the presence of a SNP to altered *in vivo *gene product function, and hence contribute to disease risk. The presence of a single nucleotide variant may perturb the function of a gene product through a range of mechanisms, including transcription factor binding; miRNA interactions; messenger RNA splicing, structure and half-life; translation efficiency; and non-synonymous substitution effects. There have been a number of approaches to the prioritization of SNPs and corresponding genes from GWAS signals using genomic locations, functional annotations, network rewiring, and integrating evidence from multiple sources [1-7]. In this study, we estimate the contribution of five molecular mechanisms falling into three major classes - missense SNPs that directly affect protein function (subdivided into those predicted to have a high impact and those predicted to have a lower impact), SNPs that alter expression level, and SNPs that may affect splicing (subdivided into those directly affecting splice sites and those acting through auxiliary splicing signals).

### Genotype/phenotype relationships in complex trait disease

The relationship between genome sequence and a complex trait disease phenotype is neither straightforward nor completely understood. Further, GWA studies, though powerful, have a number of limitations, and provide only a partial and biased view of the nature of complex trait disease. In this paper, we use the following overall model: A set of variants, differences in base sequence relative to a reference genome, each alter some aspect of the *in vivo *activity of the product of one or more genes (for convenience, we focus on protein gene products, but RNA is included in principle). In turn, these altered gene product functions change the performance of relevant pathways and processes, and these changes, together with environmental effects, affect disease relevant phenotype properties (disease risk, disease symptoms, or disease related parameters such as blood pressure or lipid levels). We refer to the variants for which there is an apparent mechanism for affecting disease phenotypes as mechanism variants, in the sense that there is some mechanism that links the presence of the variant to the change in activity of gene products and hence a disease phenotype. (Other authors have used the term 'causal SNPs', for example [8]). We are interested in elucidating the molecular level aspects of these mechanisms, where possible.

**Figure 1:**
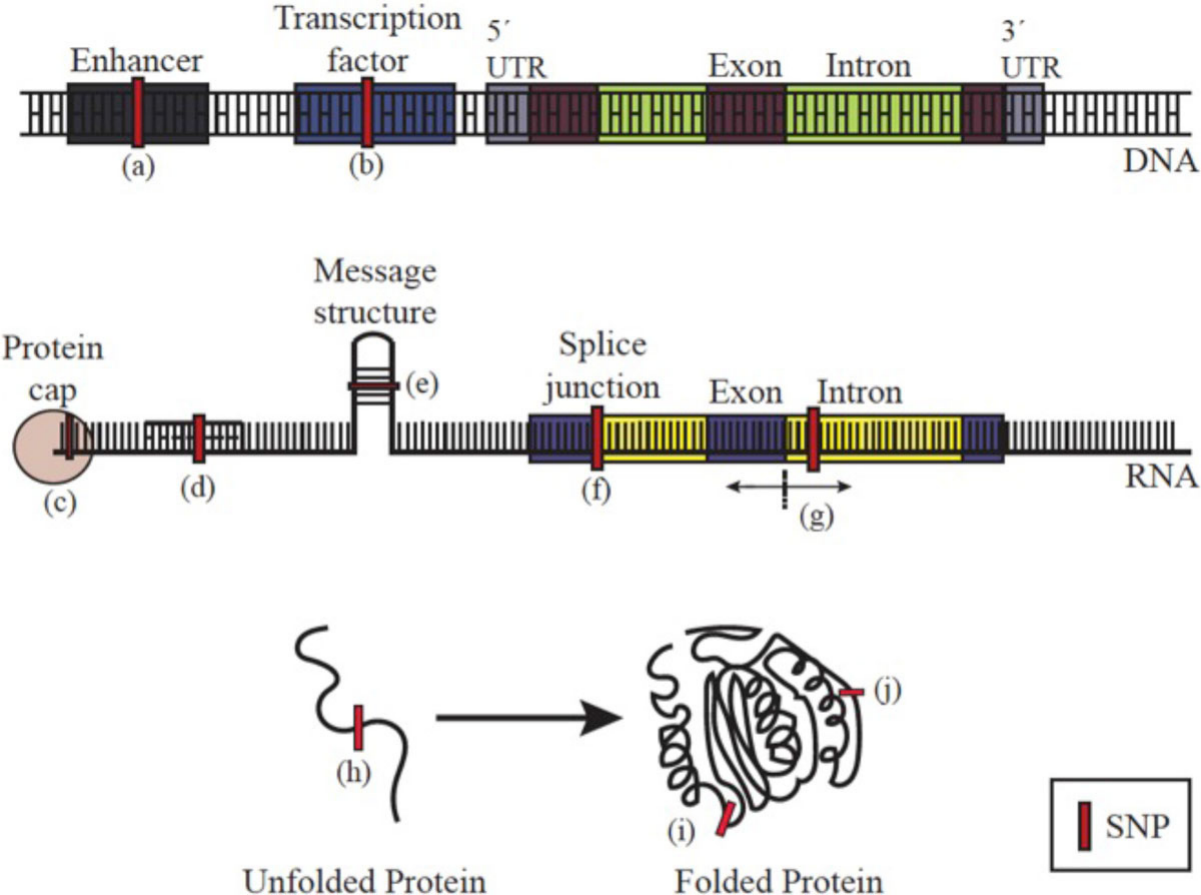
**Mechanisms by which a SNP or other genetic variant may affect the *in vivo* level of activity of a protein nearby in the DNA sequence**. At the DNA level, there may be an impact on the efficiency of transcription through effects on regulatory (a) and transcription factor (b) binding. At the RNA level, message stability and efficiency of translation may be altered by binding of protein protective factors (c) and microRNAs (d); or by a change in message structure propensities (e); or a change in sequence due to altered splicing either through direct impact on a splice junction (f) or on an auxiliary splicing signal (g). At the protein level, there may be an effect on the efficiency of folding (h) or protein half-life through destabilization of the tertiary structure (i). These variants may also cause aggregation, with toxic consequences. There may also be an effect on protein function (j), including ligand binding, catalysis, allosteric regulation, post-translational modification, and transport.

Mechanism variants may be close in DNA sequence to the affected genes, and act by such mechanisms as altering the affinity of a transcription factor for one of its binding sites, thus altering the RNA abundance for a gene; affecting the stability of a message by altering the binding of a protein or microRNA to it, affecting the rate of translation through changes in message structure propensities, affecting the distribution of splicing products by changing the affinity of a splicing modulation factor; or by resulting in an amino acid substitution that in some way affects *in vivo *function (folding, degradation, aggregation, post-translational modification, ligand binding, catalysis....). The present analysis aims to encompass all these effects. Figure 1 summarizes the mechanisms that are included.

Mechanism variants may also be remote in the sequence, acting as a consequence of the three dimensional organization of chromatin. New experimental methods such as Hi C are now providing data on chromatin three-dimensional organization [9], and cases of variants affecting the expression other than the closest gene have been found, for example [10]. Sequence remote effects act primarily on the expression level of genes, and in principle, GWAS data can be used to find associations between the presence of SNPs and the expression level of a gene wherever these occur in the genome. In practice, population sizes used in the studies limits how many associations may be tested for a single gene, since multi-testing considerations reduce the statistical significance of a particular observation. For this reason, most studies to date provide only associations between variants and genes close by in the sequence (up to 1Mb), and we restrict ourselves to these. In future, it should be possible to combine information from Hi C and similar experiments to test for associations for all SNPs that are near in space to a gene, not just in sequence.

There are also epigenetic mechanisms that alter the expression level of genes, such as patterns of DNA methylation and histone modifications. For example, a study of Type 2 Diabetes detected many locations where levels of DNA methylation are statistically different between case and control populations [11]. These differences may be inherited or environmentally induced, and to the extent that the former is true, will contribute to missing heritability from GWA studies [12]. In either case, they are outside the scope of the present analysis.

### Types of mechanism variant

Mechanism variants may be single base changes, small insertions or deletions (indels), copy number changes, or larger scale chromosome rearrangements. There are an increasing number of studies using copy number detection (for example [13]), but most GWAS results are SNP based. These studies may nevertheless identify mechanism variants other than SNPs if these are in appropriate LD with SNPs on the microarray used. In this work, for missense and splicing, we explicitly examine potential mechanism SNPs, and not other types of variant. However, in some cases, where information is derived from linked marker SNPs, such as those for disease risk and expression change, without direct identification of mechanism variants, we may also include other types of variant, particularly small indels.

### Inferring the mechanism relevance of a SNP

The involvement of a SNP in a disease related mechanism may be inferred in three basic ways. First, any effect on fitness will be reflected in the frequency and type of substitutions accepted at that position in different species. The availability of complete genome sequences for many species has allowed wide application of this approach, and it has proven effective at the amino acid level for identifying missense single base variants causative of monogenic diseases [14] and missense driver mutations in cancer [15]. We use these types of methods [14,16,17] to identify mechanism SNPs in complex trait disease. The same approach may be used at the base level to identify positions involved in mechanisms such as transcription control. There it has been less effective, although conservation of bases across a region has been used successfully to identify functional elements [18].

Secondly, direct knowledge of the functional role of DNA bases involved in a particular mechanism may be utilized. In this study, we use three types of such information: that base changes within two bases of a splice site abolish splicing at that position [19], that some base changes close to a splice site may modulate splicing at that position [20], and that missense base substitutions affect specific aspects of protein function. For the latter, we use a model that predicts effects on protein structure stability [21]. We do not make use of knowledge of functional elements at the DNA level, such as transcription sites. The ENCODE project [22] has accumulated an enormous quantity of such information. But the presence of a transcription regulatory factor binding site does not imply that binding affects transcription, and in most instances that is likely not the case [23-25].

The third class of methods for inferring a connection between a SNP and an effect on protein activity is to utilize data on an intermediate phenotype, such as mRNA abundance. So far the primary data of this type are from eQTL studies - GWA studies in which SNPs associated with a change in expression of one or more genes are identified. The phenotype of expression change may arise from a number of mechanisms at the DNA and RNA levels (Figure 1) such as an altered transcription factor or enhancer binding site, altered distribution of splicing isoforms, splicing induced nonsense mediated decay, changes in message half life arising from altered binding of factors affecting stability, altered inhibition of translation by microRNA binding, and altered rate of translation through changes in message structure propensity. The eQTL data are now very extensive, and provide a powerful means of identifying such expression related mechanisms. There are some caveats. First, reproducibility of these data has been low, implying a significant level of false positives in most single studies. We have addressed that by using only eQTL relationships found in two or more independent studies. That procedure is expected to greatly reduce false positives, but will also omit some true positives, resulting in a conservative estimate for the role of this mechanism. Second, expression related mechanisms may be tissue and population dependent. Analysis of the datasets used as the basis for this study indicates that population variability is not a major concern, but that some fraction of false positives is unavoidable when extrapolating across tissues and cell types. On very limited data, we estimate this rate at 5 - 20%; see [26] for details. Third, marker SNPs associated with an expression change must be compared with markers from the relevant GWA disease study to determine whether these are compatible with the same underlying mechanism variant.

## Results

### GWAS loci and candidate SNPs

We collected disease associated markers for 356 loci for the seven complex trait diseases included in the WTCCC1 study [27], and subsequent studies and meta-analyses of these as accessed from the GWAS catalog (Table 1). The number of identified loci varies substantially for the different diseases, from a high of 90 for Crohn's Disease to a low of 17 for Hypertension. Partly, that reflects the size of the studies that have been conducted for each disease - bigger study populations lead to the discovery of more loci. Partly this may reflect the different nature of these diseases, including the degree of genetic complexity and the role played by genetic factors not detectable by GWAS methods. Because of linkage disequilibrium, the marker SNPs in the loci represent a small fraction of total SNPs that may be involved in mechanism, and so are unlikely to be directly involved in disease mechanism, but rather are in LD with mechanism variants. For missense and splicing mechanisms, in each locus, we selected SNPs in appropriate LD to the representative marker SNP (see Methods). SNPs up to +/-200kb on either side of the marker were considered. The locus boundaries delineated by the set of accepted candidate SNPs may encompass several protein coding genes, depending on the strength of LD in the region. Details of locus boundaries are provided in supplementary material to [28]. No candidates with minor allele frequency less than 5% frequency are included, because of inadequately reliable LD estimates below that level. The process generates 235,253 candidate SNPs for the 356 included loci across the seven diseases (Table 1). These candidate SNPs form the set of variants considered for missense and splicing mechanisms. For effects on RNA expression, we compare the location of the disease marker SNPs with markers for eQTL relationships (see Methods) [26].

**Table 1:** Number of GWAS loci and candidate SNPs used in this study for seven common human diseases

Disease	Total Loci	Total candidate SNPs
Bipolar Disorder	69	37,924

Coronary Artery Disease	46	33,584

Crohn's Disease	90	57,665

Hypertension	17	12,169

Rheumatoid Arthritis	37	27,657

Type 1 Diabetes	54	34,945

Type 2 Diabetes	43	31,309

### Contributions of three primary mechanism classes in disease loci

#### Missense candidates

A total 1595 of the candidate SNPs are missense, and 69% of the 356 loci harbor at least one of these. Computational methods [14,21] were used to estimate which of these missense SNPs have a significant impact on *in vivo *protein function. A total of 432 were assigned as high-impact on this basis, providing at least one high-impact missense candidate for mechanism in 118 (33%) of the loci. A further 124 (35%) loci have a predicted low impact missense SNP and no predicted high-impact one.

#### Expression-altering candidates

A set of 16 eQTL studies (Supplementary Table 5 in Additional file 1) were matched against the disease marker SNP information for the seven diseases [26]. Only eQTL relationships observed in at least two separate studies are included. 163 (46%) of the 356 loci are found to be consistent with an underlying expression change mechanism.

#### Splicing candidates

SplicePort [20] was used to identify those SNPs likely to affect splicing efficiency through splicing signals outside of the nearly invariant GT and AG splice site dinucleotides. Applying this method to the candidate SNPs for the seven diseases, we found a set of 453 SNPs that putatively influence splicing, with at least one such SNP in 37% of the disease loci (131 loci). Supplementary Table 1 in Additional file 1 provides the scores for all tested sites. We also checked each splice junction for SNPs that directly disrupt the site. We find 37 loci (10%) out of 356 loci have at least one candidate that directly alters a splice site GT or AG dinucleotide.

### Relative roles of each mechanism in these diseases

**Figure 2:**
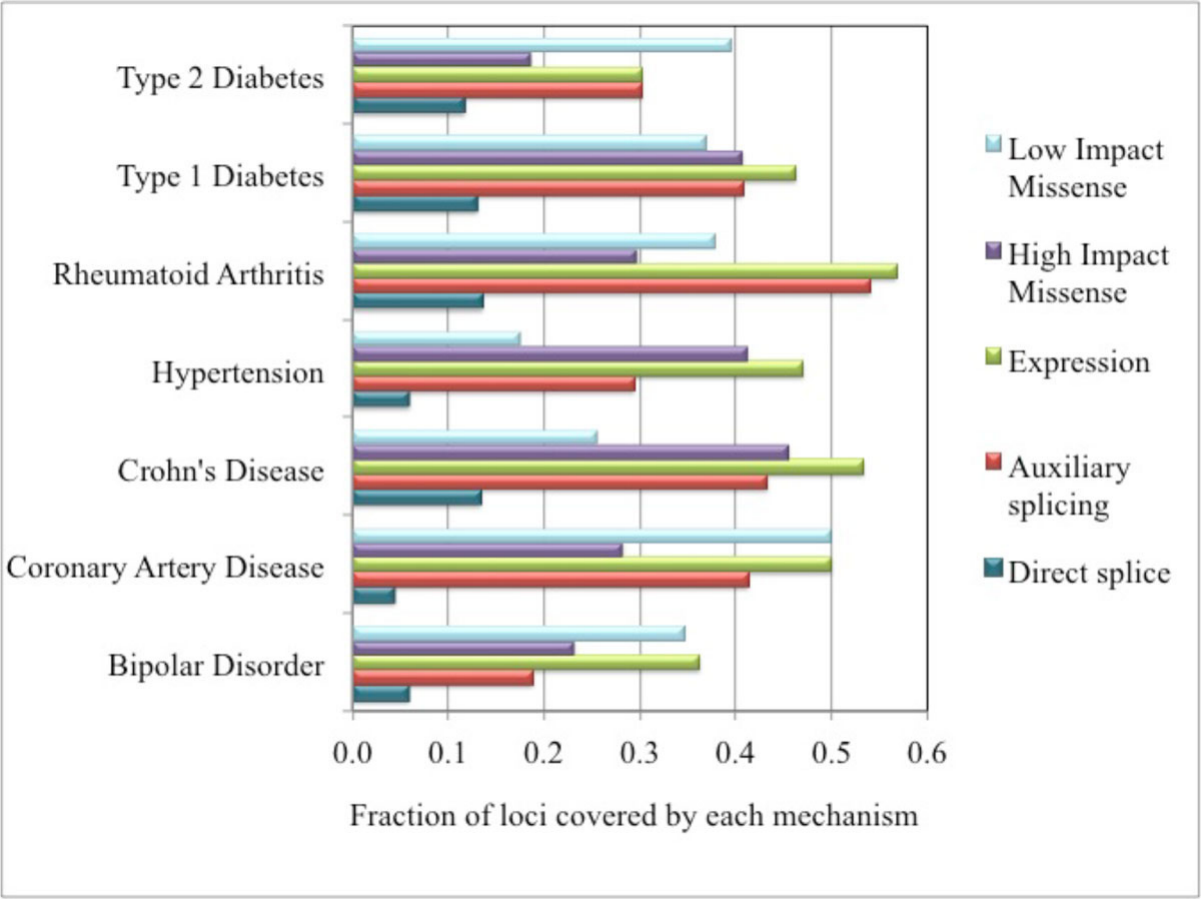
**Distribution of loci covered by each mechanism across seven diseases**.

Figure 2 shows the fraction of disease related loci in each of the seven diseases found to have a potential mechanism including high and low impact missense, expression, and splicing. Overall, 76% of these loci harbor at least one such mechanism. With the exception of direct impact on a splice site, all mechanisms occur with approximately the same frequency over the set of diseases. (Numbers for the direct splice mechanism are too low for comparison. The number of loci for Hypertension is too small for inclusion in this and the other analyses). The fraction of loci with putative mechanisms for the different diseases ranges from 85% for Coronary Artery Disease to 64% for Bipolar Disorder. In Bipolar Disorder, for expression and regulation of splicing, the low fraction of loci may partly reflect tissue specific nature of the signal, since only two heterogeneous brain samples are included in the analysis. But missense mechanisms are also lowest in this disease.

There is substantial variation in the relative roles of each mechanism within different diseases. Most notably, the ratio of low to high impact missense varies from about 2 for Coronary Artery Disease and Type 2 Diabetes, to 0.5 for Crohn's Disease, with Bipolar Disorder, Type 1 Diabetes and Rheumatoid Arthritis having intermediate ratios of about 1. A possible explanation for these striking differences is related to the fact that the diseases with a high proportion of low impact SNPs (Type 2 Diabetes and Coronary Artery Disease) are late onset, while those with lower values (Crohn's Disease, Bipolar Disorder, Type 1 Diabetes) are early onset. Mechanism variants in late onset diseases may be subject to reduced selection pressure, with the result that methods for assigning impact based on sequence profiles yield a larger proportion of variants as low impact.

Another feature of these data is that the there is a considerable variation in the relative role played by auxiliary splicing mechanisms across the diseases, from a high of 71% of Rheumatoid Arthritis loci with such mechanisms to a low of 30% for Bipolar Disorder.

### Occurrence of multiple possible mechanisms in loci

**Figure 3:**
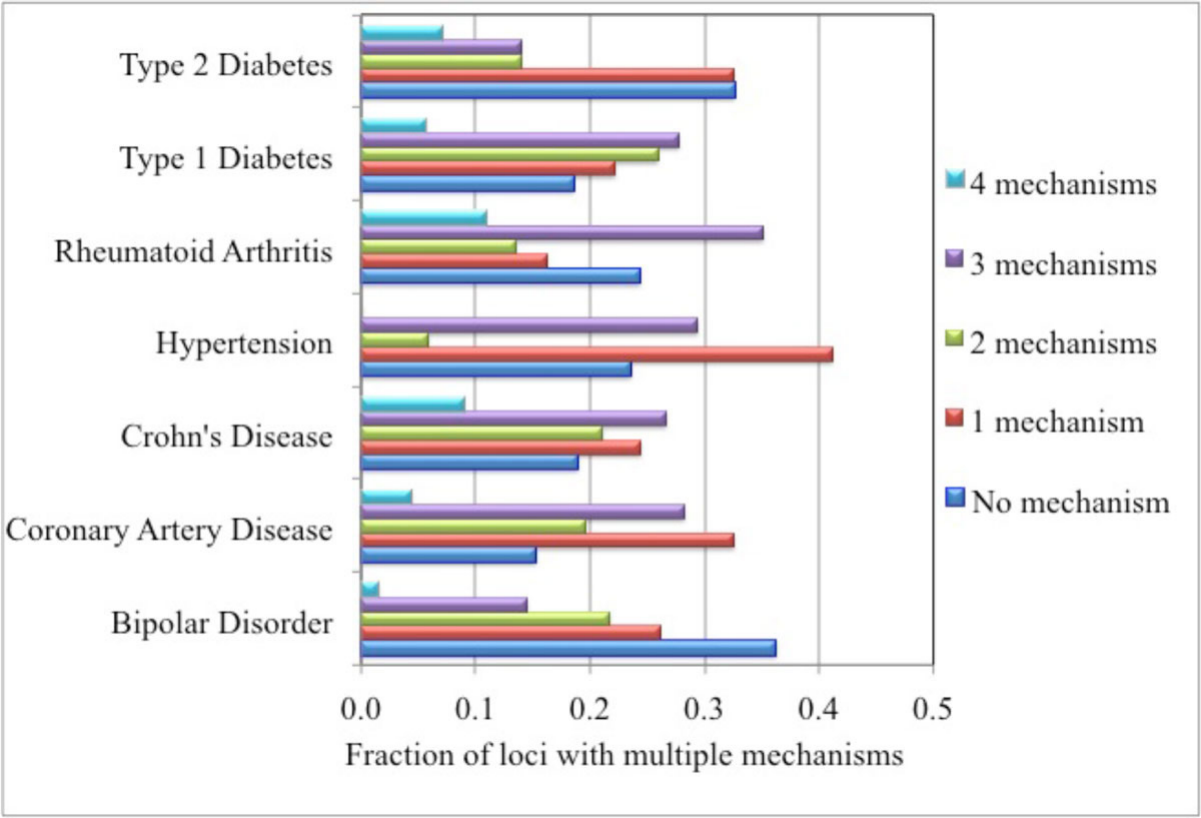
**Fraction of loci with zero, 1, 2, 3 or 4 putative mechanisms, for each of the seven diseases**.

A further question we address is the extent to which there is a single mechanism assigned each locus, or whether there is a possibility of multiple mechanisms. Figure 3 shows the distribution of number of mechanisms found per locus across the seven diseases. (A maximum of four types of mechanisms may be operative in a locus as the high-impact missense and low impact missense counts are mutually exclusive). Overall, 24% (86) of loci have no proposed mechanisms. Bipolar Disorder has the largest fraction of no mechanism (36%) and Type 2 Diabetes is also high (33%), compared to other diseases (Crohn's Disease 19%, Coronary Artery Disease 15%). For the 270 loci with at least one proposed mechanism, overall 35% have a single mechanism with the higher fractions observed for Coronary Artery Disease (38%) and Type 2 Diabetes (48%) and the lowest fraction for Rheumatoid Arthritis (21%).

Many cases of effects on splicing, both directly on splice sites and on auxiliary splice signals, are expected to be observable as expression changes, particularly as a result of nonsense mediated decay [29]. At the level of individual proteins within loci, 275 proteins have a putative splicing mechanism (including both direct splice site and auxiliary splicing sequences). Of these, 83 (30%) are also assigned an expression mechanism. Overlap is lower for other pairs of mechanisms (for example 17% of proteins with a high-impact missense mechanism also have an expression mechanism).

### Genotype/phenotype model

For present purposes, we divide the relationship between genotype and phenotype into two parts: (1) the effect of genetic variants on the activity of the relevant gene products (proteins in this model), and (2) the effect of the altered activities on the phenotype of interest. We define the total effect of a variant on a phenotype as the combination of the change in activity (A_i_) of an affected protein 'i', δA_i_, and the impact of that change on the phenotype of interest 'q', δP_iq_. δA and δP are fractional changes of activity and a phenotype parameter, such as disease risk, respectively. The relationship between the set of all altered protein activities, {δA_i_} and the total phenotype change δP_q _can be expressed as:

(1) $$\delta {\text{P}}_{\text{q}}=\text{f}\left(\left\{\delta {\text{A}}_{\text{i}}\right\},\left\{\text{E}\right\}\right)$$

where {E} is the set of relevant environmental factors. In a GWA study, environmental factors are considered to be averaged out over the population samples or to be constant, and so can be omitted. Then we can write the total effect of all perturbed gene products on the phenotype 'q' as a Taylor expansion:

(2) $$\delta P_{q}=\overset{n}{\underset{i=1}{\sum }}\frac{\partial P_{q}}{\partial A_{i}}\delta A_{i}+\overset{n}{\underset{i=1}{\sum }}\overset{n}{\underset{j=1}{\sum }}\frac{\partial^{2}P_{q}}{\partial A_{i}\partial A_{j}}\delta A_{i}\delta A_{j}+\ldots$$

where the first term represents the linear portion of the relationship between an activity changes of the gene products {δA_i_} and the phenotype, and the second and higher terms represent non-linear contributions and also epistatic effects. In GWAS data, epistatic effects are generally undetectable, probably because of the study sizes are too small to represent such higher dimensional cross terms, so data for these contributions are not available. The linear approximation to the coupling between {δA_i_} and δP_q_ may be expressed as:

(3) $$\delta {\text{P}}_{\text{q}}=\overset{n}{\underset{i=1}{\sum }}S_{iq}\delta {\text{A}}_{\text{i}}$$

where S_iq _= ∂*P_q _*/ ∂*A_i _*and is a dimensionless coefficient representing the sensitivity of phenotype 'q' to change of activity of gene 'i', equivalent to that used as a local sensitivity coefficient in system robustness analysis [30]. There are limitations to the linear model, as first identified by [31]. Errors from non-linearity will be smaller when δA is small. Here the model serves largely as qualitative approximation between the size of a protein activity perturbation and the severity of the resulting phenotypic impact.

### Properties of δA, the change in protein activity resulting from the presence of an SNP

The protein *in vivo *activity change δA may be positive or negative, representing a gain or loss of function respectively at the molecular level. eQTL studies of the relationship between the presence of an SNP and message level show an approximately equal fraction of increase versus decrease of expression [26]. Most missense changes reduce protein activity [32], and rare missense variants causing monogenic disease also usually reduce activity, often through destabilization of protein three-dimensional structure [21,33]. Similarly, in cancer, missense mutations in tumor suppressors reduce molecular activity [15], while mutations in oncogenes result in a gain of activity of some form. In any given protein, many possible missense mutations lead to loss of function, while only a few very specific ones result in gain of function. For this reason, we expect most missense SNPs contributing in complex trait disease to result in loss of molecular activity. Direct splice site hits and auxiliary splicing changes also lead to loss of function.

The size of activity change δA resulting from an SNP also varies greatly. eQTL studies show quite small expression differences associated with the presence of SNPs, with a median change just over two fold [26]. Missense variants range in impact from neutral to essentially abolishing function [32]. Monogenic disease mutations, for example those causing phenylketonuria [30], usually exhibit a very large reduction in protein activity, in excess of five fold. Computational methods are trained on these types of disease data, and so detect loss of activity greater than that magnitude. We refer to these levels of activity change as 'high-impact'. Direct splice site changes also usually result in a high impact on protein activity, while auxiliary splicing variants, like missense variants, vary in impact.

### Properties of S_iq_, the sensitivity coefficient relating change of protein activity and a disease phenotype

Subject to correction for incomplete linkage disequilibrium between marker SNPs and mechanism variants, values of δP_iq_ are known from GWAS. Generally, the S_iq_ values, reflecting the strength of coupling between protein activity and a disease phenotype, are unknown. For a few GWAS loci, where a mechanism has been identified and quantified, δA_i_ is known, and so S_iq_ can be obtained using equation 3. For example, a missense mechanism SNP in MSP (Macrophage stimulating protein) has been shown to reduce interaction with the cell surface receptor RON approximately five fold [34] thus for a homozygous substitution δA = -0.8 (80% loss of activity). This mechanism allele is in almost complete linkage disequilibrium with a Crohn's Disease marker SNP for the locus, and the odds ratio of disease in the presence of the homozygous minor genotype versus in the presence of the homozygous major genotype is 1.84 [27], corresponding to a δP of 0.84, yielding a sensitivity coefficient S, δP/δA, of 0.84/0.8, approximately 1.

Only a small fraction of all genes affect any particular disease phenotype 'q', so that for most genes, S_iq_ is essentially zero. For some others, δP_iq _is too small to be detectable in a usual GWAS experiment [35]. Many genes with a large sensitivity coefficient S are also not be discovered by GWAS, including most known drug targets [36]. (Likely because there is strong selection against variants that affect the activity of these proteins). For most genes with intermediate impact on the phenotype an association will be discovered, provided the mechanism variant is in sufficiently strong linkage disequilibrium with one or more tag SNPs on the microarray used [37]. Two factors affect whether δP_iq_ exceeds the detectability threshold - the amplitude of S_iq_ and the amplitude of δA_i_. For genes with large coupling to the phenotype (large |S_iq_|), low impact variants, such as those arising from expression, auxiliary splicing or low impact missense, may produce a sufficiently large δA to provide a detectable association with the disease phenotype. For such genes, high-impact missense SNPs or splice site hits may produce a high penetrance monogenic disease effect [38]. For genes with a small S_iq _to a particular disease phenotype 'q', low impact SNPs will not result in a sufficiently large value of δP_iq_, whereas high-impact missense or direct splice hits may be detected.

### Functional relevance of proteins with mechanism variants

The presence of a mechanism variant affecting the activity of a particular protein does not necessarily imply that the protein is involved in the disease. Particularly when there is more than one apparent mechanism in a locus affecting different proteins, one or more of these may be irrelevant (that is, the proteins concerned have a near zero sensitivity coefficient, S, to the disease phenotype). Supporting evidence of relevance can be provided by the broader biological role of an implicated protein. Here we use criteria of whether the protein has been suggested as appropriate by the authors of a GWA study, or whether there is other literature support for its relevance. We subdivide all 1014 proteins that have a putative mechanism for a specific disease into four categories of disease relevance. (Note that since some proteins have mechanisms for more than one disease, the total number of unique proteins is lower, at 840). Category A contains those proteins where the specific mechanism has been already recognized in the literature for the corresponding disease; category B is for those proteins that are already proposed as candidate proteins for involvement in disease mechanism through GWA and other studies, but for which the molecular mechanism has yet to be established; and category C contains those proteins without a previously proposed relationship with the corresponding disease. All MHC region proteins are categorized separately, as category D, as proteins in this region have a well-established connection with the immune component of the diseases concerned and extensive linkage disequilibrium makes assignment of specific mechanisms difficult.

Table 2 shows the number of proteins in each category for each mechanism, for all diseases. The higher number (24) of category A proteins for change in expression compared to other mechanisms is primarily a consequence of a number of studies that have looked at difference in expression between disease case and control populations for genes that have been implicated by GWA studies, for example [39,40]. Proteins previously identified as disease relevant have significantly more mechanisms assigned than those not previously considered disease relevant (all P values < 0.0001, see Methods), as would be expected if most proteins involved in mechanism have been correctly identified. High-impact missense and expression mechanisms occur proportionately more in classes A and B than in class C (ratios of 1.4 and 1.5 respectively), compared to low impact missense and auxiliary splicing (ratios of 0.79 and 0.96). This variation suggests a higher fraction of false positives in low impact missense and auxiliary splicing than in the other mechanisms. Note that although there is an overall enrichment of mechanisms in previously identified proteins, it is likely that some of the newly implicated proteins are also involved in the diseases.

**Table 2:** Number of proteins in each category of disease relevance for each mechanism.

Protein category	#Proteins with a low impact missense mechanism	#Proteins with a high impact missense mechanism	#Proteins with a change in expression mechanism	#Proteins with a putative splicing mechanism	#Proteins with a direct splice mechanism
A	4	3	24	3	2

B	168	104	155	109	17

C	219	77	121	116	14

D	37	42	81	58	15

Table 3 shows the number of loci in each category for each mechanism for all diseases. (In assigning categories to loci, if a locus contains proteins in more than one category, A takes precedence of B, which takes precedence over C).

**Table 3:** Number of loci in each category of disease relevance for each mechanism

Locus category	#Loci with at least 1 low impact missense mechanism	#Loci with at least 1 high impact missense mechanism	#Loci with at least 1 change in expression mechanism	#Loci with at least 1 putative splicing mechanism	#Loci with at least 1 direct splice mechanism
A	2	3	21	3	2

B	84	75	101	71	15

C	39	32	32	48	13

D	1	8	9	9	6

13% of all expression mechanism loci have a category A protein, substantially more than for other mechanisms. For all mechanisms, the relative role of class C proteins is lower here than in the protein level analysis, as a consequence of a portion of class C proteins occurring in loci where there is also a class A or B one.

**Figure 4:**
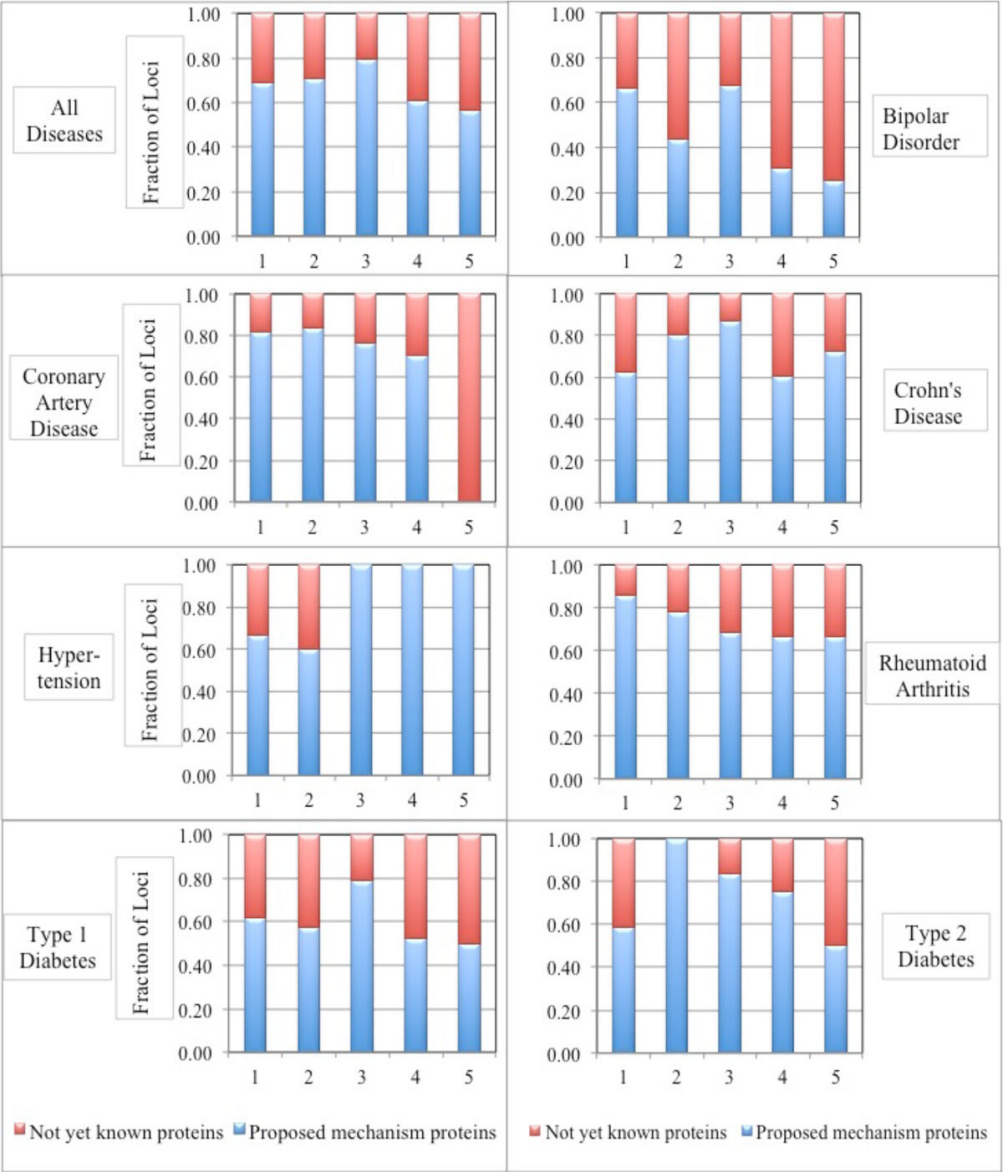
**Fraction of loci with proposed mechanism proteins (categories A and B) (blue bars) and without such proteins (red bars) for each disease**. X axis labels are: 1: Loci with a low impact msSNP; 2: Loci with a high-impact msSNP; 3: Loci with an expression mechanism; 4: Loci with an auxiliary splicing mechanism; 5: Loci with a direct splicing mechanism.

Figure 4 shows the fraction of loci with proposed mechanism proteins (categories A and B), for each mechanism, for each disease. There is considerable variation by disease. For example, the fraction of loci with candidate proteins is smaller in Bipolar Disorder than in other diseases. Variability is especially high for loci containing high-impact missense variants. For example, for Type 2 Diabetes, only seven of the 42 non-MHC loci contain at least one high-impact missense variant, and all of these have at least one category B protein. For Rheumatoid Arthritis, many category C proteins in which mechanisms occur have been identified as relevant (category B) for other autoimmune diseases such as Celiac Disease and Multiple Sclerosis, reflecting incomplete annotation. The fraction of putative auxiliary splicing mechanisms also varies across the diseases, and is lowest for Bipolar Disorder.

Supplementary Table 4 in Additional file 1 lists the mechanisms for all category A proteins.

## Methods

More extensive information on the methods used to identify putative mechanisms is given in [28](missense), [26] (expression), and [20] (http://Spliceport.org, auxiliary splicing). Here we recapitulate key aspects. Data for the missense analysis are available as supplementary information in [28], and those for the expression analysis via [26]. Splicing data are in the supplementary material in Additional file 1 for this paper.

### Collection of GWAS data

High confident GWAS markers (21 loci with P-value < 5.0x10^-7^) were collected from the Wellcome Trust Case Control Consortium (WTCCC1) [27] seven disease study (Bipolar Disorder, Coronary Artery Disease, Crohn's Disease, Hypertension, Rheumatoid Arthritis, Type 1 Diabetes, and Type 2 Diabetes), together with other significant markers (an additional 335 loci with P-value < 1E-05) from subsequent studies and meta-analyses as compiled in the GWAS catalog (http://www.genome.gov/gwastudies, accessed on September, 2013).

### Identification of candidate missense and splicing mechanism SNPs

Linkage disequilibrium (LD) data from Hapmap (hapmap release#27 - merged I+II: rel #24 and III: release #2, NCBI build 36, February 2009) and 1000genomes data (interim Phase 1 release Nov, 2011; http://www.1000genomes.org/data) were used to compile a candidate list of possible mechanism SNPs within each GWAS locus, extending out to a maximum of +/-200kb around the representative locus marker SNP. An SNP is included as a candidate if the LD relationship and frequency are such that involvement in mechanism would generate the observed case/control frequency difference for the marker [28]. To test whether this condition is fulfilled, the case/control odds ratio implied at a potential candidate SNP is calculated from the marker SNP odds ratio and the LD relationship between the two SNPs. The method was validated by comparison with frequencies imputed from full genotype data for the WTCCC1 study [27]. The set of accepted candidate SNPs define the extent of the corresponding locus. Current data provide reliable LD estimates for most candidates for with minor allele frequency ≥ 5%.

### Missense candidates

Missense annotation for the candidate SNPs was taken from dbSNP137 (http://www.ncbi.nlm.nih.gov/projects/SNP/). Likely impact of the SNPs on *in vivo *protein activity was first assessed using two computational methods (SNPs3D structure and SNPs3D profile) [14,21]. These methods use support vector machine (SVM) models, trained on monogenic disease mutations and a control set, and classify each missense SNP as high or low impact on *in vivo *protein function, where high impact corresponds to approximately a five fold or greater loss of function [32,41]. The SNPs3D structure method makes use of structural information to estimate the effect of an amino acid substitution on protein stability [21]. The SNPs3D profile method utilizes features based on sequence conservation within the protein family and the probability of occurrence of the particular amino acid substitution introduced by the base variant [14]. Two other missense analysis methods, SIFT [16] and Polyphen2 [17], were used to assess the extent of consensus in the high impact assignments. These methods also primarily use features based on observed residue preferences at the substitution position. Full details of the methods are provided in [28].

### Expression altering candidates

eQTL relationships and disease marker SNPs were compared to identify those instances where the data are compatible with a common underlying mechanism variant, taking into account LD relations. The method was calibrated using relationships between these two types of markers for situations where fuller disease SNP information was obtained by imputation. On that basis, disease marker and eQTL marker SNPs are considered to represent the same underlying expression mechanism if they are identical, or within 0.05 centiMorgans of each other. To address the issue of noise in the eQTL data, a set of consensus eQTLs was obtained by integrating 16 publicly available eQTL datasets (Supplementary Table 5 in Additional file 1). To be included in the consensus set, and eQTL must have been discovered in at least two independent studies, either with identical marker SNPs, or with marker SNPs in LD to each other at r^2 ^> 0.8. Full details of the methods are available in [26].

### Splicing candidates

SplicePort [20] (http://spliceport.org) was used to identify those SNPs likely to affect splicing efficiency. SplicePort uses a feature generation algorithm to score every AG and GT dinucleotide within 80 nucleotides on either side of each known splice site. Distinct SplicePort score change thresholds were applied for three intervals around donor sites and four intervals around acceptor sites (Supplementary Table 2 in Additional file 1). These thresholds are the median score change in a set of 184 true positives, mutations with a *bona fide *effect on splicing. All pairs of variants and splice sites for which the variant produces a SplicePort score change above the threshold were considered as candidates. Variants were from release 64 (ftp://ftp.ensembl.org/pub/release-64/variation/gvf/homo_sapiens/) and RefSeq splice sites were based on hg19. A complete list (across the genome) of pairs above these thresholds can be viewed at http://spliceport.org/trueY.html; GWAS SNPs in this set are listed in Supplemental Table 1 in Additional file 1.

SNPs falling in the following locations were considered to directly affect splicing: Within introns, the two bases at the 5' end (splice donor) and the two bases at the 3' end (splice acceptor). Within exons, the base at the 5' end (acceptor site) and the three bases at the 3' end (donor site). Supplementary Table 3 in Additional file 1 lists all the SNPs that are overlapping with direct splice sites.

### Test for mechanism enrichment

There are 1867 genes (excluding those in the MHC region) in the 356 disease loci that contain at least one qualified candidate SNP (i.e. an SNP with LD to the marker such that it could produce the observed marker case/control frequency difference), and therefore could have been assigned a mechanism. These were divided into the 555 proteins that are listed in the GWAS catalog as disease relevant, or were assigned by us a disease relevant on the basis of literature (the 'known' protein set) and the remaining 1312 proteins with no indication of disease relevance (the 'unknown' set). A two-tailed Fisher's exact test was used to determine whether the number of proteins containing each mechanism is enriched in the known set compared to that expected by chance.

## Discussion

We have examined which of five possible molecular level mechanisms may underlie a set of 356 loci where the presence of SNPs is associated with altered risk for seven common complex trait diseases. With the assumptions used, we find that a large fraction of these loci (76%) have at least one of these mechanisms present, and in many loci, there is more than one possible mechanism. The high coverage suggests that most observed GWAS associations can be explained by one of these mechanisms. A number of mechanisms are not included in this analysis. Since we only consider effects on expression caused by variants local (within 1Mb) in sequence to affected genes, effects mediated by spatial rather than sequence proximity between the variant and a gene will be omitted. Since the analysis is confined to GWAS data and variants with frequency higher than 0.05, we also omit other contributions related to missing heritability, such as rare variants [12] and variants with weak effect sizes [42].

Of the five mechanisms considered, we find the largest role for expression, but also substantial roles for three others: high and low impact missense, and auxiliary splicing. The fifth mechanism, direct hits on splice sites, is relatively infrequent, with between 4 and 22 instances per disease. The fraction of loci with a putative mechanism varies across the seven diseases, ranging from 64% to 85% of loci. Reasons for that are not clear, but it may be that particular diseases have a higher role of mechanisms not included in this study.

A number of loci have more than one mechanism assigned, some times of both high and low impact (large and small δA) at the molecular level. For instance, an effect on expression (low impact) and a high-impact missense. That does not necessarily imply that the high-impact one will dominate. In the model used here (equation 3) the impact on the phenotype, δP, is the product of impact of a mechanism at the molecular level, δA, and the coupling of the activity of that gene to the phenotype, S. In the same locus, a gene with a high-impact mechanism (large δA) maybe weakly coupled to the disease phenotype (small S), whereas a gene with a lower impact mechanism (small δA) is tightly coupled (very large S) and so dominates. As noted earlier, we have observed a significant **decrease **in high-impact missense SNPs in genes most tightly coupled to disease phenotypes (drug targets, generally having the largest S values) [36]. Presumably, this is a consequence of selection against missense variants that affect these gene products. That is, selection likely results in an inverse correlation between the size of molecular impact (δA) and coupling to the disease phenotype (S).

Within the GWAS disease loci, we observe that each mechanism is substantially enriched in proteins previously suggested as disease relevant (P < 0.00001 in all cases). The enrichment is much larger for high-impact missense and eQTL mechanisms compared to the other proteins present in the loci, suggesting these are the most reliable assignments. Least enriched is the low impact missense mechanism. The 'low impact' designation indicates that the effect size on protein activity is below some threshold, and it may lie anywhere between that threshold and zero. Those at the lower end of the range are not expected to be involved in disease mechanism, so this is an inherently very noisy category. Although enrichment of proposed mechanisms in relevant proteins is a useful indication of the validity of the analysis, some of occurrences in other proteins will also be correct.

The potential of all five molecular mechanisms to affect the *in vivo *activity of proteins occurs with relatively high frequency in the genome. The set of high confidence eQTL relationships included have at least one relationship between the presence of an SNP and altered gene expression for about 4000 genes [26], implying that on average about one in four genes with significant expression has at least one local SNP that affects its expression level. Similarly, in dbSNP137, about 1/3 of genes contain at least one missense SNP with frequency 5% or greater. There is a similarly high number of SNPs predicted to regulate alternative splicing [20]. Thus, for any gene with a significant coupling to a disease phenotype (i.e. significant S value), one or more mechanisms will likely be available. In other words, what primarily determines whether a gene is related to a complex trait phenotype is not whether there is a potential expression, coding, or splicing mechanism available - there usually will be - but whether the activity of the gene is sufficiently coupled to the phenotype (large enough S value). Consistent with that, we find no significant enrichment of eQTLs [26] or missense SNPs [28] in complex trait loci.

The role of the mechanisms in the different diseases varies. Most notably, there is a tendency for early onset diseases to exhibit a higher fraction of loci with putative high-impact missense SNPs versus low impact ones, compared with late onset diseases. It may be that mechanism variants in late onset disease are under too weak selection pressure for the sequence profile based impact assignment methods to be fully effective. Those methods depend on selection pressure influencing the type of amino acid substitutions that become fixed in each species. Theoretically, selection is not operative if |s| < 1/2N_e _[43], where 's' is the selection coefficient of a genetic variant, and Ne is the effective population size. For humans, Ne is considered to be approximately 10,000 [44], so that selection is only operative for |s| > 0.5 × 10^−4^. A typical complex disease marker is associated with an increased risk of disease of the order of 10^−2^. For early onset disease, such as Bipolar Disorder, where the disease likely affects reproductive success, this constitutes a significant selection pressure. But for late onset, such as Coronary Artery Disease, even allowing for a small fraction of early onset cases, it may be insignificant in fitness terms. The wealth of new human sequence data is beginning to allow evaluation of selection pressure at the gene level, using population genetics models. Results so far suggest weak selection effects for variants associated with some classes of complex trait disease [45], but at present have insufficient resolution to distinguish between late and early onset. Greater amounts of sequence data should provide more definitive results. Accumulation of more information on the functional role of the amino acids at each substitution site, requiring three-dimensional structure and other information, will directly allow comparison of function based assessment methods with profile ones for the same variants, also helping to resolve this question.

In the last few years there have been a number of studies aimed at using GWAS results to determine which type of mechanisms underlie complex traits. These methods typically divide the genome into zones, such as DNAase-I hypersensitive (DHS), coding, UTRs, introns and so on, and estimate the contribution of variants to traits in each of these regions, often in terms of enrichment in each region compared with expectation. Schork et al. [46] used a relatively simple SNP function assignment method based on considering the functional setting of SNPs in LD with GWAS markers and looking for category enrichment for 14 complex traits. The greatest enrichment was found in 5'UTRs, followed by exons. Intergenic regions showed the lowest signal, depleted more than 10 fold. Pickrell [47] used a model that splits the genome into blocks, each of which may contain one or no causal SNPs, and used a Bayesian model to assign probabilities of association, applying the method to 18 traits. The largest enrichments were found for non-synonymous SNPs, ranging for 4 fold to 32 fold, depending on the trait. However, because only a small fraction of SNPs are in coding regions that results in only an estimated 2 to 20% fraction of GWAS associations driven by non-synonymous SNPs. Maurano et al. [48] estimated the fraction of GWAS marker SNPs that are associated with SNPs in DHS and coding regions, finding a 40% enrichment in DHSs, and estimating that 77% of non-coding GWAS SNPs are in or close to DHS regions, while only 11% are similarly close to coding regions. Gusev et al. [49] calculated narrow sense heritability contributed by SNPs in six different functional categories for 11 common diseases, using a linear mixed model [42] to estimate the narrow sense heritability contributed by each region. This model effectively integrates over signals contributing to heritability over all SNPs in a region, not just those in GWAS identified loci, and has been found to account for a much larger fraction of heritability, suggesting many contributions have effect sizes that are too small to be detected by GWAS. They found the greatest enrichment for coding SNPs (14 fold), but the category of DHS sites, though only 5 fold enriched, contributed most heritability (79%), because of the much larger number of SNPs included, with approximately 10% contributed by coding regions. Farh et al. [50] developed a method for estimating which SNPs are most likely to be causal, based on dense genotyping data obtained using a specialized immune disease chip, and then extrapolating to other complex traits. The method uses a Bayesian model to derive the probability of each SNP being causal, given the structure and observed pattern of associations with the trait for SNPs across the locus. These authors find enrichment of non-synonymous SNPs among those most likely causal, but find that overall only 14% of the included loci have a likely non-synonymous casual SNP. They find strong enrichment of putative causal SNPs in the vicinity of enhancers, as well as cell type dependent effects. Kichaev et al. [51] integrated functional annotation with genetic association data using an empirical Bayes prior to improve prioritization of causal variants in fine mapping studies. For four lipid traits, they find increased probability of causality for variants in exons and transcription start sites, and decreased probability in local repressed chromatin. Common themes in these and other studies are greatest enrichment of trait related variants in coding regions, but because of the relatively small number of bases in that classification, a relatively small role for this class in complex traits, and with the major role for non-coding SNPs, especially in DHS or other regions relevant to expression regulation.

An alternative strategy to region enrichment methods is to identify those SNPs that alter molecular level activity and are also in appropriate linkage disequilibrium with GWAS marker SNPs. Nica et al. [52] looked for potentially causal SNPs related to both a GWAS disease marker and a eQTL SNP, and in this way identified a small set of candidate genes. Nicolae et al. [53] showed that SNPs associated with complex traits are significantly more likely to be in eQTLs than a set of random microarray SNPs with the same frequency distribution. We have used this type of SNP matching approach to identify trait related SNPs expected to alter molecular level activity by one of three explicit, local in sequence, mechanisms (missense, expression, splicing). The results are qualitatively similar to the region analysis methods in assigning the largest role to SNPs in some way involved in expression variation (34% of loci including proteins of known relevance, up to 46% total), consistent with local DHS and 5'UTR enrichment. There is a rather larger role for missense (22% of loci including proteins of known relevance, up to 33% total) compared with the values found in region analyses which range from 10 to 20% [47-50]. The reasons for the lower role for missense in these other studies are not yet clear. As noted above, there is general agreement on a greater enrichment of coding regions, showing that on a per nucleotide basis, there are more mechanisms in coding regions than elsewhere. But integrating over the larger number of SNPs included in regulatory related regions, particularly DHSs, more than offsets the enrichment effect. All the models (including ours) have a number of approximations, and the full impact of these is not yet known. Gusev et al. discuss a number of factors that may affect the performance of linear mixed models [49]. Maurano et al. [48] restrict potential coding mechanism SNPs to those in strong LD with variants within genes whereas we consider weaker LD relationships, providing these are in appropriate LD with a marker. It is usually considered that weaker LD relationships will contribute a large number of candidates [54].

We also propose a relatively high occurrence of effects on potential auxiliary splicing signals. So far, there is limited direct data on these, but they may be under-reported because they affect expression through nonsense-mediated decay or have phenotypic effects attributed to their dual identity as missense mutations [55]. We used what might be considered a conservative threshold based of 50% sensitivity in the case of known mutations in auxiliary splicing signals that affect splicing (supplementary Table 2 in Additional file 1). However, a high false positive rate is possible if there is heterogeneity between genes (or exons) in their dependence on auxiliary splicing signals (as seems likely). More benchmarking is needed to confirm the reliability of the method, and the necessary experimental data are now becoming available. Ultimately, SplicePort can be improved by using thresholds specific to each splicing event and training with data on the impact of variants on splicing rather than splice site identification. A report published after the initial submission of this work [56] using a similar splicing assessment tool, likewise predicts a surprisingly large impact of single nucleotide variants on splicing. However, many significant differences in design and application prevent direct comparisons.

At present, in all methods, because of uncertainty as to which SNPs are really causative, there will be some false assignments. In any given locus, proposed mechanisms should be regarded as hypotheses for further testing and examination of supporting data. The field is advancing rapidly as new datasets become available, including large numbers of fully sequenced genomes, expression, DNA methylation and other studies of case and control populations for each disease, detailed follow-up studies of possible mechanisms in each locus, high throughput assays to determine the effect of genetic variants on protein function (for example [57]), and improved computational methods based on detailed models of the function of each protein. Understanding of the processes and pathway linking molecular level properties to disease phenotypes is also advancing. These and other factors will result in a much clearer picture of complex trait disease emerging in the next few years.

## Abbreviations used

Genome wide association study: GWAS; Single nucleotide variant: SNV; Single nucleotide polymorphism: SNP; Human Gene Mutation Database: HGMD; Support vector machine: SVM.

## Competing interests

The authors declare they have no conflict of interests in relation to this VarI-SIG issue article.

## Authors' contributions

JM and LRP conceived this work and participated in its design. LRP performed all missense analysis and the analysis of the combined mechanisms. C-H.Y. and LRP performed the expression analysis. SMM performed the auxiliary splicing analysis. All authors contributed to the writing of the manuscript and have read and approved it.

## Supplementary Material

Additional file 1 Supplementary Table 1, Supplementary Table 2, Supplementary Table 3, Supplementary Table 4, Supplementary Table 5. Click here for file

## Declarations

The publication costs for this article were funded by a grant from the National Institutes of Health GM104436.

This article has been published as part of *BMC Genomics *Volume 16 Supplement 8, 2015: VarI-SIG 2014: Identification and annotation of genetic variants in the context of structure, function and disease. The full contents of the supplement are available online at http://www.biomedcentral.com/bmcgenomics/supplements/16/S8.
