# Supplementary material for: Insights from GWAS: emerging landscape of mechanisms underlying complex trait disease
Source: BMC Genomics. 2015 Jun 18;16(Suppl 8):S4. doi: 10.1186/1471-2164-16-S8-S4 (PMC4480957; doi:10.1186/1471-2164-16-S8-S4)
Supplement: Additional file 1 — Supplementary Table 1, Supplementary Table 2, Supplementary Table 3, Supplementary Table 4, Supplementary Table 5. [file 1471-2164-16-S8-S4-S1.pdf]

Supplementary Table 1: SNPs affecting auxiliary splicing signals, using SplicePort

Disease	SNP	Chr	SNP pos.	SS pos.	SS_type	ref_score	alt_score	diff	cutoff	gene (s)
BD	rs59271006	2	97529360	97529418	Donor_Intron_5SS	0.3324	0.443	-0.1106	0.071	SEMA4C
BD	rs6750988	2	97544089	97544152	Donor_Intron_5SS	1.5444	1.7204	-0.1761	0.071	FAM178B
BD	rs2276824	3	52637486	52637536	Donor_Intron_5SS	0.9024	0.7939	0.1085	0.071	PBRM1
BD	rs2286798	3	52821177	52821203	Acceptor_Intron_3SS	1.3262	1.4552	-0.1291	0.111	ITIH1
BD	rs1076425	3	52825462	52825532	Acceptor_Intron_3SS	-0.3957	-0.2575	-0.1382	0.111	ITIH1
BD	rs9324	3	52825585	52825643	Donor_Exon_5SS	0.9118	1.1711	-0.2593	0.18	ITIH1
BD	rs2245538	3	52852060	52852067	Donor_Intron_5SS	0.7752	0.6612	0.114	0.071	ITIH4
BD	rs2276815	3	52853747	52853769	Donor_Intron_5SS	0.2534	-0.0351	0.2886	0.071	ITIH4
BD	rs682396	3	182870082	182870162	Donor_Intron_5SS	0.5622	0.4717	0.0905	0.071	LAMP3
BD	rs10987596	9	130094526	130094552	Acceptor_Intron_3SS	-0.425	-0.5717	0.1468	0.111	GARNL3
BD	rs13292096	9	130191186	130191198	Donor_Exon_5SS	1.5638	1.0915	0.4724	0.18	ZNF79
BD	rs2244624	9	130213508	130213559	Donor_Intron_5SS	0.8461	0.9673	-0.1212	0.071	RPL12
BD	rs2243906	9	130219669	130219671	Donor_Exon_5SS	1.1467	0.9009	0.2459	0.18	LRSAM1
BD	rs2073822	9	135982088	135982134	Acceptor_Exon_3SS	1.3702	1.6237	-0.2535	0.217	RALGDS
BD	rs12804382	11	66458696	66458754	Donor_Intron_5SS	0.3607	0.5882	-0.2276	0.071	SPTBN2
BD	rs10875894	12	49314862	49314864	Donor_Exon_5SS	0.0588	0.2575	-0.1987	0.18	CCDC65
BD	rs17040655	12	108919166	108919233	Donor_Intron_5SS	0.1657	0.0655	0.1002	0.071	SART3
BD	rs6571751	14	21770730	21770742	Donor_Exon_5SS	0.596	0.8949	-0.2989	0.18	RPGRIP1
BD	rs11074559	16	23486197	23486224	Donor_Intron_5SS	1.2838	1.209	0.0748	0.071	GGA2
BD	rs722069	16	23506940	23507014	Donor_Intron_5SS	1.17	1.0767	0.0933	0.071	GGA2
BD	rs2072062	16	23540798	23540822	Donor_Intron_5SS	1.8129	1.7315	0.0814	0.071	EARS2
BD	rs2074303	19	19381755	19381830	Donor_Intron_5SS	1.4097	1.5044	-0.0947	0.071	TM6SF2
BD	rs2074550	19	19387743	19387755	Donor_Intron_5SS	1.921	1.7487	0.1723	0.071	SUGP1
BD	rs281385	19	49217261	49217301	Acceptor_Exon_3SS	0.853	1.1081	-0.2551	0.217	MAMSTR
BD	rs55966626	19	49342396	49342461	Donor_Intron_5SS	0.9322	1.0695	-0.1373	0.071	PLEKHA4
BD	rs17272658	19	49355405	49355484	Donor_Intron_5SS	0.8018	0.9551	-0.1533	0.071	PLEKHA4
BD	rs73063523	19	49408943	49409009	Acceptor_Intron_3SS	0.8052	0.9558	-0.1506	0.111	NUCB1
BD	rs709012	20	3675498	3675569	Acceptor_Exon_3SS	1.0537	0.7918	0.2619	0.217	SIGLEC1
CAD	rs585131	1	55524116	55524171	Acceptor_Intron_3SS	1.2898	1.4018	-0.1119	0.111	PCSK9
CAD	rs487230	1	55541174	55541195	Acceptor_Exon_3SS	1.9643	2.2259	-0.2616	0.217	USP24
CAD	rs548852	1	55555285	55555299	Donor_Intron_5SS	0.6606	0.8353	-0.1747	0.071	USP24

CAD	rs4970834	1	109814880	109814899	Acceptor_BPS_3SS	0.9916	1.2067	-0.215	0.186	CELSR2
CAD	rs611917	1	109815252	109815261	Acceptor_Ytract_3SS	0.4824	-0.0661	0.5486	0.367	CELSR2
CAD	rs629001	1	109838918	109838991	Acceptor_Exon_3SS	0.0056	-0.2147	0.2203	0.217	MYBPHL
CAD	rs2228604	1	109884775	109884779	Acceptor_Exon_3SS	0.765	1.0201	-0.2551	0.217	SORT1
CAD	rs11142	1	109897103	109897152	Acceptor_Exon_3SS	0.7717	1.0458	-0.2741	0.217	SORT1
CAD	rs6740918	2	234093035	234093053	Acceptor_BPS_3SS	1.8592	2.0574	-0.1982	0.186	INPP5D
CAD	rs2241880	2	234183368	234183423	Donor_Exon_5SS	0.7741	0.5745	0.1996	0.18	ATG16L1
CAD	rs6789076	3	15807725	15807740	Donor_Intron_5SS	0.7181	0.829	-0.1109	0.071	ANKRD28
CAD	rs2233974	6	31080016	31080067	Acceptor_Exon_3SS	2.1124	1.8374	0.275	0.217	C6orf15
CAD	rs3130559	6	31097301	31097345	Acceptor_Intron_3SS	-1.0277	-0.8532	-0.1745	0.111	PSORS1C1
CAD	rs2233945	6	31107361	31107417	Acceptor_Intron_3SS	0.0581	0.2266	-0.1686	0.111	PSORS1C1
CAD	rs130073	6	31111180	31111189	Acceptor_Exon_3SS	0.7605	1.0672	-0.3067	0.217	CCHCR1
CAD	rs3094225	6	31113052	31113078	Acceptor_Exon_3SS	2.126	2.4586	-0.3326	0.217	CCHCR1
CAD	rs45438402	6	31133663	31133703	Donor_Intron_5SS	-0.3013	-0.5112	0.2098	0.071	POU5F1
CAD	rs72856758	6	31141954	31142014	Donor_Intron_5SS	0.6827	0.5748	0.1079	0.071	PSORS1C3
CAD	rs3094189	6	31141970	31142014	Donor_Intron_5SS	0.745	0.5936	0.1514	0.071	PSORS1C3
CAD	rs9264594	6	31237048	31237114	Donor_Intron_5SS	1.2506	1.335	-0.0845	0.071	HLA-C
CAD	rs9264609	6	31237664	31237742	Donor_Intron_5SS	0.2579	0.379	-0.1211	0.071	HLA-C
CAD	rs17884362	6	31237917	31237986	Donor_Intron_5SS	0.7697	1.0628	-0.2931	0.071	HLA-C
CAD	rs17886232	6	31237965	31237986	Donor_Intron_5SS	1.1984	1.0934	0.105	0.071	HLA-C
CAD	rs9264653	6	31238814	31238849	Donor_Intron_5SS	0.595	0.6863	-0.0913	0.071	HLA-C
CAD	rs17879195	6	31239305	31239375	Donor_Intron_5SS	2.3539	2.2523	0.1016	0.071	HLA-C
CAD	rs7383157	6	31239306	31239375	Donor_Intron_5SS	2.3777	2.2523	0.1254	0.071	HLA-C
CAD	rs9264672	6	31239766	31239775	Donor_Intron_5SS	0.2274	0.3939	-0.1665	0.071	HLA-C
CAD	rs9264674	6	31239868	31239854	Acceptor_Ytract_3SS	-0.7446	-0.0749	-0.6697	0.367	HLA-C
CAD	rs2074491	6	31239896	31239854	Acceptor_Intron_3SS	-0.1645	-0.2841	0.1196	0.111	HLA-C
CAD	rs1054111	6	31322026	31322072	Acceptor_Exon_3SS	0.8723	0.4345	0.4378	0.217	HLA-B
CAD	rs3819305	6	31322175	31322255	Donor_Intron_5SS	1.7659	1.8375	-0.0716	0.071	HLA-B
CAD	rs17199328	6	31322395	31322441	Acceptor_Exon_3SS	0.9536	1.179	-0.2254	0.217	HLA-B
CAD	rs9266193	6	31324788	31324862	Donor_Intron_5SS	1.4404	1.5724	-0.132	0.071	HLA-B
CAD	rs9266198	6	31324847	31324862	Donor_Intron_5SS	1.429	1.3032	0.1258	0.071	HLA-B
CAD	rs2234045	6	35088381	35088422	Acceptor_Exon_3SS	0.6984	1.0695	-0.3711	0.217	TCP11
CAD	rs2234044	6	35090097	35090113	Acceptor_Exon_3SS	1.0143	1.4495	-0.4352	0.217	TCP11
CAD	rs10276604	7	107216738	107216810	Acceptor_Intron_3SS	0.4094	0.5451	-0.1357	0.111	DUS4L

CAD	rs3124747	9	136268084	136268140	Donor_Exon_5SS	0.8409	1.0997	-0.2588	0.18	C9orf96
CAD	rs28510482	9	136268912	136268951	Donor_Exon_5SS	0.3225	-0.0096	0.3321	0.18	C9orf96
CAD	rs2285485	9	136277381	136277418	Donor_Intron_5SS	0.485	0.5693	-0.0843	0.071	REXO4
CAD	rs3780807	9	136279432	136279458	Acceptor_Intron_3SS	-3.1991	-3.3257	0.1265	0.111	REXO4
CAD	rs3124765	9	136328657	136328676	Donor_Exon_5SS	1.5607	1.9996	-0.4389	0.18	C9orf7
CAD	rs304504	10	91137826	91137896	Donor_Exon_5SS	1.8522	2.1781	-0.3259	0.18	IFIT1B
CAD	rs3740393	10	104636655	104636710	Acceptor_Intron_3SS	1.1586	1.0394	0.1193	0.111	C10orf32-AS3MT,AS3MT
CAD	rs3736922	10	104850632	104850692	Donor_Intron_5SS	0.7812	0.7041	0.0772	0.071	NT5C2
CAD	rs1926030	10	104855656	104855695	Donor_Intron_5SS	-0.3142	-0.4105	0.0963	0.071	NT5C2
CAD	rs3741301	11	116631391	116631450	Donor_Intron_5SS	0.752	0.6147	0.1374	0.071	BUD13
CAD	rs5092	11	116693464	116693500	Acceptor_Exon_3SS	0.1993	-0.0257	0.225	0.217	APOA4
CAD	rs695029	11	126174164	126174175	Donor_Exon_5SS	-0.5171	-0.3358	-0.1812	0.18	DCPS
CAD	rs637462	11	126176578	126176638	Donor_Exon_5SS	0.7581	0.9927	-0.2346	0.18	DCPS
CAD	rs651922	11	126201290	126201299	Acceptor_Ytract_3SS	0.5939	0.116	0.4779	0.367	DCPS
CAD	rs17525809	12	121592689	121592755	Donor_Exon_5SS	2.0742	2.3096	-0.2354	0.18	P2RX7
CAD	rs7132846	12	121614934	121614949	Acceptor_Ytract_3SS	1.2521	1.6617	-0.4096	0.367	P2RX7
CAD	rs3743075	15	78909452	78909474	Acceptor_Exon_3SS	0.1683	0.4113	-0.243	0.217	CHRNA3
CAD	rs7161774	15	79069734	79069785	Donor_Intron_5SS	1.5025	1.3157	0.1868	0.071	ADAMTS7
CAD	rs10519215	15	79183747	79183825	Acceptor_Intron_3SS	0.1692	-0.0497	0.2188	0.111	MORF4L1
CAD	rs1836556	15	79185877	79185880	Acceptor_Ytract_3SS	1.1731	0.6278	0.5453	0.367	MORF4L1
CAD	rs12438433	15	79189282	79189324	Acceptor_Intron_3SS	2.3992	2.1809	0.2183	0.111	MORF4L1
CAD	rs12148472	15	79231478	79231512	Acceptor_Exon_3SS	0.2609	0.6147	-0.3538	0.217	CTSH
CAD	rs8076897	17	1985050	1985103	Donor_Intron_5SS	0.5135	0.6113	-0.0978	0.071	SMG6
CAD	rs2124344	17	17480195	17480233	Donor_Intron_5SS	2.2889	2.1509	0.138	0.071	PEMT
CAD	rs3818717	17	17707105	17707162	Donor_Exon_5SS	1.7683	2.0542	-0.2858	0.18	RAI1
CAD	rs3887854	17	46868710	46868739	Donor_Intron_5SS	-0.1704	-0.087	-0.0834	0.071	TTLL6
CAD	rs1962412	17	46970259	46970271	Donor_Exon_5SS	-1.2577	-1.0576	-0.2001	0.18	ATP5G1
CAD	rs1058018	17	47000251	47000298	Donor_Exon_5SS	2.2165	1.9638	0.2527	0.18	UBE2Z
CAD	rs2270576	17	47007963	47007973	Acceptor_Exon_3SS	2.2454	1.9919	0.2535	0.217	SNF8
CAD	rs2291725	17	47039132	47039180	Acceptor_Exon_3SS	0.4909	0.7659	-0.2749	0.217	GIP
CAD	rs8078510	17	47045862	47045878	Donor_Intron_5SS	-0.1856	-0.2786	0.093	0.071	GIP
CAD	rs7275	19	11170839	11170862	Donor_Exon_5SS	0.4589	0.8784	-0.4196	0.18	SMARCA4
CAD	rs754528	19	11305245	11305265	Acceptor_Exon_3SS	1.4624	1.7066	-0.2442	0.217	KANK2

CAD	rs3747137	22	26879691	26879639	Donor_Exon_5SS	0.6924	0.8761	-0.1837	0.18	HPS4
CD	rs1039863	1	78481640	78481697	Acceptor_Intron_3SS	1.3118	1.4448	-0.133	0.111	DNAJB4
CD	rs17031795	1	114255828	114255900	Donor_Intron_5SS	0.5587	0.4068	0.1519	0.071	PHTF1
CD	rs1230640	1	114267347	114267380	Donor_Intron_5SS	-0.6292	-0.5176	-0.1116	0.071	PHTF1
CD	rs2075569	1	155209360	155209406	Donor_Intron_5SS	1.0365	1.1314	-0.0949	0.071	GBA
CD	rs2297222	1	160969110	160969185	Donor_Intron_5SS	1.3585	1.4892	-0.1307	0.071	F11R
CD	rs790056	1	160969585	160969665	Donor_Intron_5SS	1.0634	0.9763	0.0871	0.071	F11R
CD	rs2297220	1	160971015	160971074	Donor_Intron_5SS	-0.1799	-0.036	-0.1439	0.071	F11R
CD	rs2292095	1	200826395	200826433	Acceptor_Intron_3SS	-0.144	-0.2875	0.1434	0.111	CAMSAP2
CD	rs1554286	1	206944233	206944251	Donor_Intron_5SS	1.8255	1.7516	0.074	0.071	IL10
CD	rs1518111	1	206944645	206944700	Donor_Intron_5SS	1.6604	1.7665	-0.1061	0.071	IL10
CD	rs2304426	2	25602142	25602158	Donor_Intron_5SS	2.1293	1.99	0.1393	0.071	DTNB
CD	rs12475426	2	27599132	27599179	Acceptor_Intron_3SS	0.9464	0.8186	0.1278	0.111	SNX17
CD	rs780106	2	27681598	27681645	Donor_Intron_5SS	1.3566	1.261	0.0956	0.071	IFT172
CD	rs813592	2	27721971	27722008	Acceptor_Intron_3SS	-0.2858	-0.1257	-0.1601	0.111	GCKR
CD	rs1177295	2	61308517	61308586	Acceptor_Intron_3SS	-0.2873	-0.4287	0.1414	0.111	KIAA1841
CD	rs4988955	2	102967928	102967995	Acceptor_Intron_3SS	0.6626	0.5353	0.1273	0.111	IL1RL1
CD	rs1035130	2	103001402	103001457	Donor_Exon_5SS	0.2522	0.0515	0.2007	0.18	IL18R1
CD	rs13018234	2	231036866	231036889	Acceptor_Exon_3SS	0.4866	0.1821	0.3045	0.217	SP110
CD	rs7590429	2	231258150	231258175	Donor_Exon_5SS	-0.3963	-0.2005	-0.1958	0.18	SP140L
CD	rs2241880	2	234183368	234183423	Donor_Exon_5SS	0.7741	0.5745	0.1996	0.18	ATG16L1
CD	rs4855839	3	49507847	49507865	Donor_Exon_5SS	1.0384	1.2459	-0.2074	0.18	DAG1
CD	rs9858542	3	49701983	49701986	Donor_Exon_5SS	1.1422	1.434	-0.2917	0.18	BSN
CD	rs9822268	3	49719729	49719780	Acceptor_Intron_3SS	1.4124	1.1377	0.2747	0.111	APEH
CD	rs34614773	3	49753003	49753007	Acceptor_Ytract_3SS	0.3642	-0.0934	0.4576	0.367	RNF123
CD	rs13316065	3	49884913	49884957	Donor_Intron_5SS	0.7465	0.8642	-0.1177	0.071	TRAIP
CD	rs62260755	3	49898318	49898375	Donor_Intron_5SS	0.6679	0.577	0.0909	0.071	CAMKV
CD	rs26503	5	96078309	96078358	Acceptor_Intron_3SS	1.3786	1.2311	0.1476	0.111	CAST
CD	rs26505	5	96079387	96079449	Donor_Exon_5SS	0.2523	-0.0976	0.3498	0.18	CAST
CD	rs27654	5	96093222	96093286	Acceptor_Intron_3SS	0.0291	-0.12	0.1491	0.111	CAST
CD	rs61745685	5	96117555	96117557	Acceptor_Exon_3SS	-0.0276	-0.3139	0.2863	0.217	ERAP1
CD	rs17482078	5	96118866	96118938	Acceptor_Exon_3SS	-0.1955	-0.4943	0.2988	0.217	ERAP1
CD	rs26618	5	96130836	96130864	Acceptor_Exon_3SS	1.6688	2.0949	-0.4261	0.217	ERAP1
CD	rs2549782	5	96231000	96231062	Donor_Exon_5SS	0.1032	-0.1948	0.2979	0.18	ERAP2

CD	rs2549795	5	96244638	96244664	Acceptor_Intron_3SS	1.4265	1.3052	0.1213	0.111	ERAP2
CD	rs2292260	5	131606936	131606995	Acceptor_Intron_3SS	2.0847	1.9653	0.1194	0.111	PDLIM4
CD	rs272893	5	131663062	131663095	Donor_Exon_5SS	1.4918	1.8347	-0.3429	0.18	SLC22A4,LOC553103
CD	rs2070728	5	131819977	131820053	Donor_Intron_5SS	1.2742	1.1365	0.1377	0.071	IRF1
CD	rs10214312	5	131821866	131821942	Donor_Intron_5SS	0.4265	0.5546	-0.1282	0.071	IRF1
CD	rs9282761	5	131822224	131822248	Donor_Intron_5SS	1.254	1.3307	-0.0767	0.071	IRF1
CD	rs4959787	6	3264502	3264558	Donor_Exon_5SS	0.6336	0.9022	-0.2686	0.18	PSMG4
CD	rs2233974	6	31080016	31080067	Acceptor_Exon_3SS	2.1124	1.8374	0.275	0.217	C6orf15
CD	rs3130559	6	31097301	31097345	Acceptor_Intron_3SS	-1.0277	-0.8532	-0.1745	0.111	PSORS1C1
CD	rs2233945	6	31107361	31107417	Acceptor_Intron_3SS	0.0581	0.2266	-0.1686	0.111	PSORS1C1
CD	rs130073	6	31111180	31111189	Acceptor_Exon_3SS	0.7605	1.0672	-0.3067	0.217	CCHCR1
CD	rs3094225	6	31113052	31113078	Acceptor_Exon_3SS	2.126	2.4586	-0.3326	0.217	CCHCR1
CD	rs3094189	6	31141970	31142014	Donor_Intron_5SS	0.745	0.5936	0.1514	0.071	PSORS1C3
CD	rs9264594	6	31237048	31237114	Donor_Intron_5SS	1.2506	1.335	-0.0845	0.071	HLA-C
CD	rs9264609	6	31237664	31237742	Donor_Intron_5SS	0.2579	0.379	-0.1211	0.071	HLA-C
CD	rs17884362	6	31237917	31237986	Donor_Intron_5SS	0.7697	1.0628	-0.2931	0.071	HLA-C
CD	rs17886232	6	31237965	31237986	Donor_Intron_5SS	1.1984	1.0934	0.105	0.071	HLA-C
CD	rs9264653	6	31238814	31238849	Donor_Intron_5SS	0.595	0.6863	-0.0913	0.071	HLA-C
CD	rs17879195	6	31239305	31239375	Donor_Intron_5SS	2.3539	2.2523	0.1016	0.071	HLA-C
CD	rs7383157	6	31239306	31239375	Donor_Intron_5SS	2.3777	2.2523	0.1254	0.071	HLA-C
CD	rs9264672	6	31239766	31239775	Donor_Intron_5SS	0.2274	0.3939	-0.1665	0.071	HLA-C
CD	rs9264674	6	31239868	31239854	Acceptor_Ytract_3SS	-0.7446	-0.0749	-0.6697	0.367	HLA-C
CD	rs2074491	6	31239896	31239854	Acceptor_Intron_3SS	-0.1645	-0.2841	0.1196	0.111	HLA-C
CD	rs3819305	6	31322175	31322255	Donor_Intron_5SS	1.7659	1.8375	-0.0716	0.071	HLA-B
CD	rs17199328	6	31322395	31322441	Acceptor_Exon_3SS	0.9536	1.179	-0.2254	0.217	HLA-B
CD	rs9266193	6	31324788	31324862	Donor_Intron_5SS	1.4404	1.5724	-0.132	0.071	HLA-B
CD	rs41557213	6	31324819	31324862	Donor_Intron_5SS	1.5128	1.4374	0.0754	0.071	HLA-B
CD	rs9266198	6	31324847	31324862	Donor_Intron_5SS	1.429	1.3032	0.1258	0.071	HLA-B
CD	rs9266824	6	31382770	31382788	Acceptor_BPS_3SS	1.7192	1.387	0.3323	0.186	MICA
CD	rs2286713	6	31473844	31473919	Acceptor_Intron_3SS	-1.0976	-1.2586	0.161	0.111	MICB
CD	rs16889320	6	159414899	159414957	Acceptor_Exon_3SS	1.203	0.9623	0.2406	0.217	RSPH3
CD	rs9459780	6	167271632	167271687	Donor_Intron_5SS	0.843	0.6683	0.1747	0.071	RPS6KA2
CD	rs2236312	6	167360104	167360169	Donor_Intron_5SS	0.3469	0.4311	-0.0842	0.071	RNASET2

CD	rs10951145	7	26778406	26778413	Donor_Intron_5SS	0.3543	0.2553	0.099	0.071	SKAP2
CD	rs45592338	9	139257422	139257440	Donor_Intron_5SS	1.2606	1.053	0.2076	0.071	DNLZ
CD	rs3812573	9	139276680	139276704	Donor_Intron_5SS	1.7519	1.8564	-0.1045	0.071	SNAPC4
CD	rs1051957	9	139298593	139298645	Acceptor_Exon_3SS	0.4087	0.6902	-0.2815	0.217	SDCCAG3
CD	rs3087886	9	139301583	139301596	Donor_Intron_5SS	0.5012	0.6348	-0.1336	0.071	SDCCAG3
CD	rs11145967	9	139312972	139313006	Acceptor_Intron_3SS	0.9784	0.7108	0.2675	0.111	PMPCA
CD	rs10870160	9	139312996	139313006	Acceptor_Ytract_3SS	0.9029	1.4157	-0.5128	0.367	PMPCA
CD	rs3136618	10	6001696	6001716	Donor_Intron_5SS	2.055	2.1266	-0.0717	0.071	IL15RA
CD	rs108499	11	61547237	61547313	Acceptor_Intron_3SS	0.9363	1.2158	-0.2796	0.111	C11orf9
CD	rs509360	11	61548559	61548609	Acceptor_Intron_3SS	0.208	0.0774	0.1306	0.111	C11orf9
CD	rs174533	11	61549025	61549044	Acceptor_BPS_3SS	0.14	0.5231	-0.3832	0.186	C11orf9
CD	rs174535	11	61551356	61551396	Donor_Exon_5SS	0.7266	0.966	-0.2395	0.18	C11orf9
CD	rs2070232	11	63970625	63970699	Donor_Exon_5SS	1.0936	1.3112	-0.2176	0.18	STIP1
CD	rs11603538	11	63986713	63986722	Acceptor_Ytract_3SS	1.7552	1.3355	0.4197	0.367	FERMT3
CD	rs1059440	11	63991801	63991812	Acceptor_Exon_3SS	0.0806	0.366	-0.2854	0.217	TRPT1
CD	rs3751440	13	44596756	44596803	Donor_Exon_5SS	0.8088	1.0613	-0.2524	0.18	LINC00284
CD	rs366615	14	88411837	88411896	Donor_Intron_5SS	1.5777	1.6894	-0.1118	0.071	GALC
CD	rs12888666	14	88431898	88431972	Acceptor_Exon_3SS	0.421	0.6649	-0.2439	0.217	GALC
CD	rs9282861	16	28617514	28617556	Acceptor_Exon_3SS	0.4883	0.2041	0.2842	0.217	SULT1A1
CD	rs4149389	16	28618011	28618081	Donor_Intron_5SS	1.2076	1.1282	0.0794	0.071	SULT1A1
CD	rs34954534	16	28618018	28618081	Donor_Intron_5SS	1.0766	1.2705	-0.1939	0.071	SULT1A1
CD	rs28410083	16	28631360	28631383	Donor_Intron_5SS	0.7804	0.665	0.1155	0.071	SULT1A1
CD	rs2305479	17	38062217	38062237	Acceptor_Exon_3SS	0.5502	0.2034	0.3468	0.217	GSDMB
CD	rs1011082	17	38068514	38068578	Donor_Intron_5SS	2.1396	2.0297	0.1099	0.071	GSDMB
CD	rs7212944	17	38122686	38122689	Donor_Exon_5SS	1.1615	1.3648	-0.2034	0.18	GSDMA
CD	rs9916279	17	38146154	38146181	Donor_Exon_5SS	0.8824	1.2781	-0.3957	0.18	PSMD3
CD	rs2302774	17	38183090	38183108	Donor_Intron_5SS	0.691	0.5051	0.1859	0.071	MED24
CD	rs7502514	17	38188844	38188901	Donor_Intron_5SS	0.9824	0.8915	0.091	0.071	MED24
CD	rs1474040	17	40722162	40722200	Donor_Exon_5SS	0.8765	1.0696	-0.1931	0.18	MLX
CD	rs2301810	19	984533	984562	Donor_Exon_5SS	0.6548	0.9112	-0.2564	0.18	WDR18
CD	rs2074573	19	985815	985864	Acceptor_Intron_3SS	-0.1147	0.0187	-0.1334	0.111	WDR18
CD	rs2240147	19	989730	989761	Acceptor_Intron_3SS	2.7738	3.0088	-0.235	0.111	WDR18
CD	rs2240149	19	990819	990851	Acceptor_Intron_3SS	0.6957	0.547	0.1487	0.111	WDR18
CD	rs8178977	19	1106477	1106538	Acceptor_Intron_3SS	1.1803	1.0159	0.1644	0.111	GPX4

CD	rs11669443	19	1109215	1109290	Donor_Intron_5SS	1.5437	1.4507	0.093	0.071	SBNO2
CD	rs4807050	19	1271529	1271549	Acceptor_BPS_3SS	1.1241	1.3262	-0.2021	0.186	CIRBP
CD	rs12488	19	1272040	1272049	Donor_Exon_5SS	1.8028	2.3099	-0.5071	0.18	CIRBP
CD	rs1056538	19	10402938	10402997	Donor_Exon_5SS	0.7162	0.9591	-0.2429	0.18	ICAM5
CD	rs3181049	19	10441117	10441135	Donor_Intron_5SS	0.5702	0.7724	-0.2022	0.071	RAVER1
CD	rs11085735	19	10602180	10602252	Donor_Intron_5SS	1.9086	2.0234	-0.1148	0.071	KEAP1
CD	rs1981827	19	33655144	33655170	Donor_Exon_5SS	0.4122	0.1145	0.2977	0.18	WDR88
CD	rs281385	19	49217261	49217301	Acceptor_Exon_3SS	0.853	1.1081	-0.2551	0.217	MAMSTR
CD	rs55966626	19	49342396	49342461	Donor_Intron_5SS	0.9322	1.0695	-0.1373	0.071	PLEKHA4
CD	rs17272658	19	49355405	49355484	Donor_Intron_5SS	0.8018	0.9551	-0.1533	0.071	PLEKHA4
CD	rs2256814	20	62373983	62374051	Acceptor_Intron_3SS	0.7772	0.4806	0.2967	0.111	SLC2A4RG
CD	rs2239573	21	34638859	34638818	Donor_Intron_5SS	0.7452	0.6273	0.1179	0.071	IL10RB
CD	rs2834167	21	34640788	34640821	Donor_Exon_5SS	0.4872	0.7005	-0.2133	0.18	IL10RB
CD	rs17883129	21	34805197	34805177	Donor_Intron_5SS	1.1252	1.322	-0.1968	0.071	IFNGR2
CD	rs2248820	21	34837623	34837648	Donor_Intron_5SS	0.9128	0.8027	0.1102	0.071	TMEM50B
CD	rs2834232	21	34892896	34892868	Acceptor_Intron_3SS	-0.2346	-0.0877	-0.1469	0.111	GART
CD	rs2834233	21	34893344	34893321	Acceptor_BPS_3SS	0.0603	-0.2271	0.2874	0.186	GART
CD	rs2834234	21	34894623	34894588	Acceptor_Intron_3SS	1.6475	1.5343	0.1132	0.111	GART
CD	rs2834236	21	34939390	34939462	Acceptor_Intron_3SS	1.6938	1.498	0.1958	0.111	SON
CD	rs1057037	21	45733867	45733903	Donor_Exon_5SS	-0.0277	-0.2666	0.2389	0.18	PFKL
CD	rs12165769	22	30399000	30398981	Donor_Intron_5SS	2.5305	2.4062	0.1243	0.071	MTMR3
CD	rs41157	22	30405151	30405117	Donor_Intron_5SS	0.9913	0.9134	0.0779	0.071	MTMR3
CD	rs41164	22	30412465	30412516	Acceptor_Intron_3SS	0.7589	0.877	-0.1181	0.111	MTMR3
CD	rs41168	22	30418161	30418127	Donor_Intron_5SS	0.4408	0.5685	-0.1277	0.071	MTMR3
CD	rs9608885	22	30738161	30738188	Donor_Intron_5SS	2.0832	1.9412	0.142	0.071	SF3A1
CD	rs5753080	22	30738737	30738793	Donor_Intron_5SS	0.5163	0.6109	-0.0946	0.071	SF3A1
CD	rs7285530	22	39710664	39710690	Donor_Intron_5SS	2.0027	1.884	0.1187	0.071	RPL3
CD	rs139472	22	41617318	41617290	Donor_Intron_5SS	0.601	0.6911	-0.0901	0.071	L3MBTL2
HT	rs12049572	1	113140583	113140592	Donor_Intron_5SS	2.3315	2.4225	-0.0911	0.071	ST7L
HT	rs2306940	1	113241052	113241099	Donor_Exon_5SS	1.0886	0.8012	0.2874	0.18	MOV10
HT	rs3013431	1	113242488	113242515	Acceptor_Intron_3SS	1.3506	1.4702	-0.1197	0.111	MOV10
HT	rs41266801	6	26197493	26197496	Acceptor_Exon_3SS	-0.1711	0.1106	-0.2817	0.217	HIST1H3D
HT	rs2286713	6	31473844	31473919	Acceptor_Intron_3SS	-1.0976	-1.2586	0.161	0.111	MICB
HT	rs3093980	6	31497478	31497523	Acceptor_Intron_3SS	1.0855	1.2488	-0.1633	0.111	MCCD1

HT	rs3093978	6	31498497	31498555	Donor_Intron_5SS	0.3307	0.4934	-0.1627	0.071	DDX39B,ATP6V1G2-DDX39B
HT	rs1129640	6	31506624	31506631	Acceptor_Exon_3SS	-0.484	-0.1871	-0.2969	0.217	DDX39B,ATP6V1G2-DDX39B
HT	rs2523502	6	31513864	31513885	Donor_Intron_5SS	0.8504	0.7791	0.0713	0.071	ATP6V1G2-DDX39B,ATP6V1G2
HT	rs2229094	6	31540556	31540617	Donor_Exon_5SS	-0.1878	0.0609	-0.2487	0.18	LTA
HT	rs2256974	6	31555392	31555417	Acceptor_BPS_3SS	-0.3426	-0.6117	0.2691	0.186	LST1
HT	rs3135041	6	31555657	31555720	Acceptor_Intron_3SS	1.2047	1.3777	-0.173	0.111	LST1
HT	rs3130071	6	31594628	31594638	Donor_Exon_5SS	0.8717	0.6619	0.2098	0.18	PRRC2A
HT	rs2736171	6	31595487	31595541	Acceptor_Intron_3SS	1.7	1.5167	0.1833	0.111	PRRC2A
HT	rs2242657	6	31602489	31602529	Acceptor_Intron_3SS	0.7183	0.5837	0.1346	0.111	PRRC2A
HT	rs11229	6	31603770	31603825	Donor_Exon_5SS	1.9375	2.1724	-0.235	0.18	PRRC2A
HT	rs3130628	6	31609272	31609315	Donor_Intron_5SS	0.5947	0.5171	0.0776	0.071	BAG6
HT	rs755714	6	31609813	31609888	Donor_Intron_5SS	0.1742	0.2805	-0.1063	0.071	BAG6
HT	rs760293	6	31611777	31611858	Donor_Intron_5SS	0.2968	0.1808	0.116	0.071	BAG6
HT	rs2242656	6	31614102	31614170	Donor_Intron_5SS	0.6281	0.725	-0.0969	0.071	BAG6
HT	rs3131383	6	31704294	31704340	Acceptor_Exon_3SS	1.808	2.0675	-0.2595	0.217	CLIC1
HT	rs3117575	6	31726253	31726324	Acceptor_Intron_3SS	1.2679	1.4088	-0.1409	0.111	MSH5
HT	rs17207524	6	31726850	31726873	Acceptor_BPS_3SS	-0.2745	-0.0703	-0.2042	0.186	MSH5
HT	rs915651	6	31749184	31749257	Donor_Intron_5SS	-0.1576	-0.3787	0.2211	0.071	VARS
HT	rs17381270	12	90015270	90015325	Donor_Intron_5SS	1.2663	1.191	0.0753	0.071	ATP2B1
HT	rs2071382	15	91428197	91428266	Acceptor_Intron_3SS	-0.1912	-0.0614	-0.1298	0.111	FES
HT	rs12909056	15	91459475	91459485	Donor_Exon_5SS	0.3441	0.7432	-0.3991	0.18	MAN2A2
HT	rs2001216	15	91498130	91498177	Donor_Exon_5SS	-0.8994	-0.6369	-0.2624	0.18	RCCD1
HT	rs2290202	15	91512267	91512308	Donor_Intron_5SS	-0.3979	-0.2978	-0.1002	0.071	PRC1
RA	rs2296442	1	2430617	2430668	Donor_Exon_5SS	0.9248	0.7011	0.2238	0.18	PLCH2
RA	rs4870	1	2488153	2488171	Donor_Exon_5SS	0.4798	0.7187	-0.2388	0.18	LOC100133445,TNFRSF14
RA	rs745368	1	2524205	2524271	Donor_Intron_5SS	1.1143	1.2204	-0.106	0.071	MMEL1
RA	rs10890216	1	38442547	38442555	Donor_Intron_5SS	1.2517	1.1327	0.1191	0.071	SF3A3
RA	rs1230640	1	114267347	114267380	Donor_Intron_5SS	-0.6292	-0.5176	-0.1116	0.071	PHTF1
RA	rs1177295	2	61308517	61308586	Acceptor_Intron_3SS	-0.2873	-0.4287	0.1414	0.111	KIAA1841
RA	rs4851287	2	100915772	100915780	Acceptor_Exon_3SS	-0.5867	-0.0397	-0.547	0.217	LONRF2

RA	rs11123823	2	100916315	100916379	Acceptor_Exon_3SS	-1.0924	-0.8083	-0.2841	0.217	LONRF2
RA	rs13317835	3	58383275	58383331	Acceptor_Intron_3SS	2.0965	2.3496	-0.2531	0.111	PXK
RA	rs56384862	3	58395863	58395885	Donor_Exon_5SS	1.0623	0.8178	0.2444	0.18	PXK
RA	rs316800	4	25316886	25316939	Acceptor_Intron_3SS	0.7975	0.9382	-0.1407	0.111	ZCCHC4
RA	rs315675	4	25363901	25363922	Donor_Exon_5SS	0.5835	0.7874	-0.2039	0.18	ZCCHC4
RA	rs10023139	4	25404553	25404592	Acceptor_Intron_3SS	0.7104	0.8837	-0.1733	0.111	ANAPC4
RA	rs26821	5	102520400	102520444	Donor_Exon_5SS	0.5867	0.8597	-0.2729	0.18	PPIP5K2
RA	rs376319	6	29644633	29644657	Donor_Intron_5SS	0.8524	0.5865	0.2659	0.071	ZFP57
RA	rs3817826	6	29696118	29696141	Donor_Intron_5SS	-0.0075	-0.1213	0.1138	0.071	HLA-F-AS1
RA	rs2735051	6	29705688	29705742	Donor_Intron_5SS	0.6622	0.5564	0.1057	0.071	HLA-F-AS1
RA	rs1629329	6	29795768	29795823	Acceptor_Intron_3SS	0.1062	0.2263	-0.1201	0.111	HLA-G
RA	rs1624337	6	29796306	29796319	Acceptor_Ytract_3SS	-0.4703	-1.2809	0.8105	0.367	HLA-G
RA	rs1632937	6	29797553	29797592	Acceptor_Intron_3SS	0.3292	0.2022	0.127	0.111	HLA-G
RA	rs3128879	6	29855682	29855731	Acceptor_Intron_3SS	-0.5444	-0.4141	-0.1303	0.111	HLA-H
RA	rs7755851	6	29857050	29857104	Acceptor_Intron_3SS	-0.2756	-0.1246	-0.1511	0.111	HLA-H
RA	rs3115603	6	29858213	29858215	Acceptor_Core_5SS	-1.0568	0.9957	-2.0525	1.455	HLA-H
RA	rs3115603	6	29858218	29858261	Donor_Exon_5SS	1.5553	1.7798	-0.2245	0.18	HLA-H
RA	rs9260155	6	29911243	29911319	Donor_Exon_5SS	-0.3186	-0.1169	-0.2017	0.18	HLA-A
RA	rs2571381	6	29912830	29912835	Acceptor_Ytract_3SS	0.3467	0.841	-0.4943	0.367	HLA-A
RA	rs112716963	6	32485815	32485830	Donor_Intron_5SS	0.2228	0.3744	-0.1517	0.071	HLA-DRB5
RA	rs114281696	6	32525925	32525962	Acceptor_Exon_3SS	-0.1267	0.126	-0.2527	0.217	HLA-DRB6
RA	rs28732249	6	32548011	32548046	Acceptor_Exon_3SS	0.7548	1.0684	-0.3136	0.217	HLA-DRB1
RA	rs9269799	6	32549313	32549333	Donor_Intron_5SS	0.0301	-0.051	0.0811	0.071	HLA-DRB1
RA	rs17885011	6	32552127	32552154	Acceptor_Exon_3SS	0.1435	0.3663	-0.2228	0.217	HLA-DRB1
RA	rs1129735	6	32605295	32605316	Donor_Exon_5SS	0.6839	0.9002	-0.2163	0.18	HLA-DQA1
RA	rs9272736	6	32609671	32609748	Acceptor_Intron_3SS	-0.2291	-0.5078	0.2788	0.111	HLA-DQA1
RA	rs9272742	6	32609722	32609748	Acceptor_Intron_3SS	-0.419	-0.2543	-0.1647	0.111	HLA-DQA1
RA	rs3188642	6	32610553	32610560	Donor_Exon_5SS	1.6227	1.8339	-0.2111	0.18	HLA-DQA1
RA	rs41270915	6	32629692	32629743	Donor_Intron_5SS	0.7741	0.6743	0.0998	0.071	HLA-DQB1
RA	rs9273912	6	32629737	32629743	Donor_Intron_5SS	0.7386	0.9475	-0.2089	0.071	HLA-DQB1
RA	rs28746795	6	32632534	32632574	Donor_Intron_5SS	1.1146	1.1909	-0.0763	0.071	HLA-DQB1
RA	rs28746798	6	32632558	32632574	Donor_Intron_5SS	1.1683	1.0107	0.1577	0.071	HLA-DQB1
RA	rs28746846	6	32634261	32634275	Donor_Intron_5SS	1.5809	1.3917	0.1891	0.071	HLA-DQB1
RA	rs3213484	6	32725062	32725080	Acceptor_Exon_3SS	0.9443	0.6691	0.2751	0.217	HLA-DQB2

RA	rs16870880	6	32782018	32782096	Donor_Intron_5SS	-0.302	-0.2163	-0.0857	0.071	HLA-DOB
RA	rs2228396	6	32797809	32797865	Acceptor_Exon_3SS	1.4959	1.1429	0.353	0.217	TAP2
RA	rs2071481	6	32819865	32819885	Donor_Intron_5SS	-0.4185	-0.3258	-0.0927	0.071	TAP1
RA	rs2236312	6	167360104	167360169	Donor_Intron_5SS	0.3469	0.4311	-0.0842	0.071	RNASET2
RA	rs2249804	8	11215617	11215644	Acceptor_Intron_3SS	0.515	0.7687	-0.2538	0.111	TDH
RA	rs2293859	8	11216761	11216792	Donor_Exon_5SS	1.0807	1.2797	-0.199	0.18	TDH
RA	rs2245250	8	11400680	11400732	Acceptor_Intron_3SS	2.1866	2.0246	0.162	0.111	BLK
RA	rs3816668	8	11406593	11406630	Donor_Exon_5SS	0.7885	1.145	-0.3566	0.18	BLK
RA	rs41274865	9	34647802	34647828	Acceptor_Intron_3SS	-1.2363	-1.0101	-0.2262	0.111	GALT
RA	rs7026635	9	123550027	123550046	Donor_Intron_5SS	2.3033	2.1243	0.179	0.071	FBXW2
RA	rs10985064	9	123626311	123626326	Donor_Intron_5SS	0.2891	0.1218	0.1673	0.071	PHF19
RA	rs17611	9	123769200	123769254	Acceptor_Exon_3SS	0.6752	0.4322	0.2429	0.217	C5
RA	rs10985126	9	123783934	123783971	Acceptor_Exon_3SS	0.1109	-0.1362	0.2471	0.217	C5
RA	rs10818503	9	123850770	123850820	Donor_Exon_5SS	0.0408	0.2723	-0.2314	0.18	CNTRL
RA	rs3136618	10	6001696	6001716	Donor_Intron_5SS	2.055	2.1266	-0.0717	0.071	IL15RA
RA	rs2301551	12	58124534	58124573	Donor_Intron_5SS	0.8548	0.7244	0.1305	0.071	AGAP2
RA	rs2069502	12	58144665	58144705	Donor_Intron_5SS	0.6803	0.7741	-0.0938	0.071	CDK4
RA	rs2305479	17	38062217	38062237	Acceptor_Exon_3SS	0.5502	0.2034	0.3468	0.217	GSDMB
RA	rs1011082	17	38068514	38068578	Donor_Intron_5SS	2.1396	2.0297	0.1099	0.071	GSDMB
RA	rs7212944	17	38122686	38122689	Donor_Exon_5SS	1.1615	1.3648	-0.2034	0.18	GSDMA
RA	rs9916279	17	38146154	38146181	Donor_Exon_5SS	0.8824	1.2781	-0.3957	0.18	PSMD3
RA	rs2302774	17	38183090	38183108	Donor_Intron_5SS	0.691	0.5051	0.1859	0.071	MED24
RA	rs7502514	17	38188844	38188901	Donor_Intron_5SS	0.9824	0.8915	0.091	0.071	MED24
RA	rs16990951	20	44578674	44578707	Acceptor_Exon_3SS	0.5645	0.3087	0.2558	0.217	ZNF335
RA	rs225358	21	43786512	43786519	Donor_Intron_5SS	2.2024	2.3049	-0.1025	0.071	TFF1
RA	rs1893592	21	43855067	43855063	Donor_Core_5SS	1.589	0.4501	1.1389	1.104	UBASH3A
RA	rs17114930	21	43857602	43857597	Acceptor_Exon_3SS	0.8814	0.4815	0.3999	0.217	UBASH3A
RA	rs117385282	21	43896143	43896156	Acceptor_Exon_3SS	1.0042	0.6533	0.3509	0.217	RSPH1
RA	rs62215906	21	43938465	43938386	Acceptor_Exon_3SS	0.5724	0.3527	0.2197	0.217	SLC37A1
RA	rs228953	22	37531436	37531367	Donor_Exon_5SS	1.7008	1.4556	0.2452	0.18	IL2RB
RA	rs228953	22	37531436	37531481	Acceptor_Exon_3SS	0.8523	0.512	0.3403	0.217	IL2RB
RA	rs229520	22	37578807	37578774	Acceptor_Intron_3SS	1.9238	2.0864	-0.1626	0.111	C1QTNF6
RA	rs229521	22	37578852	37578774	Acceptor_Intron_3SS	1.9238	1.7837	0.1401	0.111	C1QTNF6
T1D	rs1230640	1	114267347	114267380	Donor_Intron_5SS	-0.6292	-0.5176	-0.1116	0.071	PHTF1

T1D	rs1554286	1	206944233	206944251	Donor_Intron_5SS	1.8255	1.7516	0.074	0.071	IL10
T1D	rs1518111	1	206944645	206944700	Donor_Intron_5SS	1.6604	1.7665	-0.1061	0.071	IL10
T1D	rs4851287	2	100915772	100915780	Acceptor_Exon_3SS	-0.5867	-0.0397	-0.547	0.217	LONRF2
T1D	rs11123823	2	100916315	100916379	Acceptor_Exon_3SS	-1.0924	-0.8083	-0.2841	0.217	LONRF2
T1D	rs2241809	2	101014363	101014369	Donor_Intron_5SS	-0.5905	-0.3835	-0.2071	0.071	CHST10
T1D	rs17574	2	162929979	162929995	Acceptor_Exon_3SS	-0.2101	0.0175	-0.2276	0.217	DPP4
T1D	rs112716963	6	32485815	32485830	Donor_Intron_5SS	0.2228	0.3744	-0.1517	0.071	HLA-DRB5
T1D	rs114281696	6	32525925	32525962	Acceptor_Exon_3SS	-0.1267	0.126	-0.2527	0.217	HLA-DRB6
T1D	rs28732249	6	32548011	32548046	Acceptor_Exon_3SS	0.7548	1.0684	-0.3136	0.217	HLA-DRB1
T1D	rs16822516	6	32552092	32552154	Acceptor_Exon_3SS	0.1315	0.3706	-0.2391	0.217	HLA-DRB1
T1D	rs1129735	6	32605295	32605316	Donor_Exon_5SS	0.6839	0.9002	-0.2163	0.18	HLA-DQA1
T1D	rs9272736	6	32609671	32609748	Acceptor_Intron_3SS	-0.2291	-0.5078	0.2788	0.111	HLA-DQA1
T1D	rs9272741	6	32609702	32609748	Acceptor_Intron_3SS	-0.197	-0.3107	0.1137	0.111	HLA-DQA1
T1D	rs9272742	6	32609722	32609748	Acceptor_Intron_3SS	-0.419	-0.2543	-0.1647	0.111	HLA-DQA1
T1D	rs3188642	6	32610553	32610560	Donor_Exon_5SS	1.6227	1.8339	-0.2111	0.18	HLA-DQA1
T1D	rs28400568	6	32610557	32610560	Donor_Exon_5SS	1.7758	2.0142	-0.2384	0.18	HLA-DQA1
T1D	rs41270915	6	32629692	32629743	Donor_Intron_5SS	0.7741	0.6743	0.0998	0.071	HLA-DQB1
T1D	rs28746798	6	32632558	32632574	Donor_Intron_5SS	1.1683	1.0107	0.1577	0.071	HLA-DQB1
T1D	rs9274373	6	32632567	32632574	Donor_Intron_5SS	1.2689	1.1875	0.0814	0.071	HLA-DQB1
T1D	rs9274514	6	32634243	32634275	Donor_Intron_5SS	1.2601	1.1527	0.1074	0.071	HLA-DQB1
T1D	rs28746846	6	32634261	32634275	Donor_Intron_5SS	1.5809	1.3917	0.1891	0.071	HLA-DQB1
T1D	rs7774954	6	32724189	32724242	Acceptor_Exon_3SS	1.4549	1.2347	0.2202	0.217	HLA-DQB2
T1D	rs2228396	6	32797809	32797865	Acceptor_Exon_3SS	1.4959	1.1429	0.353	0.217	TAP2
T1D	rs10951145	7	26778406	26778413	Donor_Intron_5SS	0.3543	0.2553	0.099	0.071	SKAP2
T1D	rs2296441	10	100144782	100144823	Acceptor_Exon_3SS	1.0511	0.7529	0.2982	0.217	PYROXD2
T1D	rs2296438	10	100146895	100146957	Donor_Intron_5SS	0.9133	1.0928	-0.1795	0.071	PYROXD2
T1D	rs4345897	10	100147060	100147063	Acceptor_Exon_3SS	0.536	0.8124	-0.2765	0.217	PYROXD2
T1D	rs11189595	10	100167322	100167338	Donor_Intron_5SS	0.53	0.4292	0.1008	0.071	PYROXD2
T1D	rs2069398	12	56360876	56360907	Donor_Exon_5SS	0.9183	1.3049	-0.3866	0.18	CDK2
T1D	rs2271192	12	56531154	56531156	Donor_Exon_5SS	1.3875	1.5811	-0.1936	0.18	ESYT1
T1D	rs7161774	15	79069734	79069785	Donor_Intron_5SS	1.5025	1.3157	0.1868	0.071	ADAMTS7
T1D	rs10519215	15	79183747	79183825	Acceptor_Intron_3SS	0.1692	-0.0497	0.2188	0.111	MORF4L1
T1D	rs1836556	15	79185877	79185880	Acceptor_Ytract_3SS	1.1731	0.6278	0.5453	0.367	MORF4L1
T1D	rs12438433	15	79189282	79189324	Acceptor_Intron_3SS	2.3992	2.1809	0.2183	0.111	MORF4L1

T1D	rs12148472	15	79231478	79231512	Acceptor_Exon_3SS	0.2609	0.6147	-0.3538	0.217	CTSH
T1D	rs737008	16	11374866	11374891	Acceptor_Exon_3SS	1.4781	1.7519	-0.2738	0.217	PRM1
T1D	rs1827711	16	20482368	20482394	Acceptor_Intron_3SS	-0.2114	-0.3815	0.1702	0.111	ACSM2A
T1D	rs9282861	16	28617514	28617556	Acceptor_Exon_3SS	0.4883	0.2041	0.2842	0.217	SULT1A1
T1D	rs4149389	16	28618011	28618081	Donor_Intron_5SS	1.2076	1.1282	0.0794	0.071	SULT1A1
T1D	rs34954534	16	28618018	28618081	Donor_Intron_5SS	1.0766	1.2705	-0.1939	0.071	SULT1A1
T1D	rs28410083	16	28631360	28631383	Donor_Intron_5SS	0.7804	0.665	0.1155	0.071	SULT1A1
T1D	rs1109342	16	75445675	75445722	Donor_Intron_5SS	0.6394	0.8266	-0.1872	0.071	CFDP1
T1D	rs1565817	17	7700712	7700717	Acceptor_Ytract_3SS	0.6203	0.1036	0.5166	0.367	DNAH2
T1D	rs9908139	17	7830610	7830661	Donor_Intron_5SS	0.6536	0.5707	0.0829	0.071	KCNAB3
T1D	rs2305479	17	38062217	38062237	Acceptor_Exon_3SS	0.5502	0.2034	0.3468	0.217	GSDMB
T1D	rs1011082	17	38068514	38068578	Donor_Intron_5SS	2.1396	2.0297	0.1099	0.071	GSDMB
T1D	rs7212944	17	38122686	38122689	Donor_Exon_5SS	1.1615	1.3648	-0.2034	0.18	GSDMA
T1D	rs9916279	17	38146154	38146181	Donor_Exon_5SS	0.8824	1.2781	-0.3957	0.18	PSMD3
T1D	rs2302774	17	38183090	38183108	Donor_Intron_5SS	0.691	0.5051	0.1859	0.071	MED24
T1D	rs7502514	17	38188844	38188901	Donor_Intron_5SS	0.9824	0.8915	0.091	0.071	MED24
T1D	rs6416908	17	38858029	38858102	Donor_Intron_5SS	1.0963	0.9995	0.0968	0.071	KRT24
T1D	rs17558532	17	38935876	38935951	Donor_Intron_5SS	2.2748	2.1691	0.1056	0.071	KRT27
T1D	rs17558560	17	38936659	38936707	Acceptor_Exon_3SS	1.7565	1.5367	0.2198	0.217	KRT27
T1D	rs763361	18	67531642	67531674	Acceptor_Exon_3SS	1.3175	1.8782	-0.5607	0.217	CD226
T1D	rs1056538	19	10402938	10402997	Donor_Exon_5SS	0.7162	0.9591	-0.2429	0.18	ICAM5
T1D	rs3181049	19	10441117	10441135	Donor_Intron_5SS	0.5702	0.7724	-0.2022	0.071	RAVER1
T1D	rs4804134	19	10568519	10568554	Acceptor_Intron_3SS	1.6838	1.9221	-0.2383	0.111	PDE4A
T1D	rs11085735	19	10602180	10602252	Donor_Intron_5SS	1.9086	2.0234	-0.1148	0.071	KEAP1
T1D	rs3826709	19	10670184	10670204	Acceptor_Exon_3SS	2.3771	2.665	-0.2879	0.217	KRI1
T1D	rs2243603	20	1546911	1546912	Acceptor_Exon_3SS	0.1784	0.4127	-0.2342	0.217	SIRPB1
T1D	rs225358	21	43786512	43786519	Donor_Intron_5SS	2.2024	2.3049	-0.1025	0.071	TFF1
T1D	rs1893592	21	43855067	43855063	Donor_Core_5SS	1.589	0.4501	1.1389	1.104	UBASH3A
T1D	rs17114930	21	43857602	43857597	Acceptor_Exon_3SS	0.8814	0.4815	0.3999	0.217	UBASH3A
T1D	rs117385282	21	43896143	43896156	Acceptor_Exon_3SS	1.0042	0.6533	0.3509	0.217	RSPH1
T1D	rs62215906	21	43938465	43938386	Acceptor_Exon_3SS	0.5724	0.3527	0.2197	0.217	SLC37A1
T1D	rs12165769	22	30399000	30398981	Donor_Intron_5SS	2.5305	2.4062	0.1243	0.071	MTMR3
T1D	rs41157	22	30405151	30405117	Donor_Intron_5SS	0.9913	0.9134	0.0779	0.071	MTMR3
T1D	rs41164	22	30412465	30412516	Acceptor_Intron_3SS	0.7589	0.877	-0.1181	0.111	MTMR3

T1D	rs41168	22	30418161	30418127	Donor_Intron_5SS	0.4408	0.5685	-0.1277	0.071	MTMR3
T1D	rs9608885	22	30738161	30738188	Donor_Intron_5SS	2.0832	1.9412	0.142	0.071	SF3A1
T1D	rs5753080	22	30738737	30738793	Donor_Intron_5SS	0.5163	0.6109	-0.0946	0.071	SF3A1
T1D	rs228953	22	37531436	37531367	Donor_Exon_5SS	1.7008	1.4556	0.2452	0.18	IL2RB
T1D	rs228953	22	37531436	37531481	Acceptor_Exon_3SS	0.8523	0.512	0.3403	0.217	IL2RB
T1D	rs229520	22	37578807	37578774	Acceptor_Intron_3SS	1.9238	2.0864	-0.1626	0.111	C1QTNF6
T1D	rs229521	22	37578852	37578774	Acceptor_Intron_3SS	1.9238	1.7837	0.1401	0.111	C1QTNF6
T2D	rs1801282	3	12393125	12393172	Donor_Exon_5SS	1.0693	1.3372	-0.2678	0.18	PPARG
T2D	rs6762208	3	185331165	185331195	Donor_Exon_5SS	1.3224	1.002	0.3204	0.18	SENP2
T2D	rs10010131	4	6292915	6292923	Acceptor_Ytract_3SS	0.2009	-0.4134	0.6143	0.367	WFS1
T2D	rs2306419	4	122592663	122592701	Donor_Intron_5SS	0.4835	0.8145	-0.331	0.071	ANXA5
T2D	rs3217773	4	122739181	122739198	Donor_Intron_5SS	1.0383	1.207	-0.1687	0.071	CCNA2
T2D	rs3217772	4	122739183	122739198	Donor_Intron_5SS	1.2778	1.1292	0.1486	0.071	CCNA2
T2D	rs1507994	4	122749541	122749556	Donor_Intron_5SS	0.8938	0.9772	-0.0834	0.071	BBS7
T2D	rs6824258	4	122769967	122769998	Donor_Intron_5SS	0.7757	0.7021	0.0736	0.071	BBS7
T2D	rs1507993	4	122831281	122831308	Donor_Intron_5SS	1.8717	1.783	0.0886	0.071	TRPC3
T2D	rs115333128	6	32485804	32485830	Donor_Intron_5SS	0.2544	0.0426	0.2118	0.071	HLA-DRB5
T2D	rs112716963	6	32485815	32485830	Donor_Intron_5SS	0.2228	0.3744	-0.1517	0.071	HLA-DRB5
T2D	rs3828831	6	32521117	32521175	Acceptor_Exon_3SS	1.1217	1.4496	-0.3279	0.217	HLA-DRB6
T2D	rs75424216	6	32522412	32522466	Donor_Intron_5SS	0.2739	0.3615	-0.0876	0.071	HLA-DRB6
T2D	rs35570280	6	32522434	32522466	Donor_Intron_5SS	0.1823	0.0701	0.1122	0.071	HLA-DRB6
T2D	rs114281696	6	32525925	32525962	Acceptor_Exon_3SS	-0.1267	0.126	-0.2527	0.217	HLA-DRB6
T2D	rs28732249	6	32548011	32548046	Acceptor_Exon_3SS	0.7548	1.0684	-0.3136	0.217	HLA-DRB1
T2D	rs16822516	6	32552092	32552154	Acceptor_Exon_3SS	0.1315	0.3706	-0.2391	0.217	HLA-DRB1
T2D	rs1129735	6	32605295	32605316	Donor_Exon_5SS	0.6839	0.9002	-0.2163	0.18	HLA-DQA1
T2D	rs9272736	6	32609671	32609748	Acceptor_Intron_3SS	-0.2291	-0.5078	0.2788	0.111	HLA-DQA1
T2D	rs9272742	6	32609722	32609748	Acceptor_Intron_3SS	-0.419	-0.2543	-0.1647	0.111	HLA-DQA1
T2D	rs3188642	6	32610553	32610560	Donor_Exon_5SS	1.6227	1.8339	-0.2111	0.18	HLA-DQA1
T2D	rs9273912	6	32629737	32629743	Donor_Intron_5SS	0.7386	0.9475	-0.2089	0.071	HLA-DQB1
T2D	rs28746795	6	32632534	32632574	Donor_Intron_5SS	1.1146	1.1909	-0.0763	0.071	HLA-DQB1
T2D	rs28746798	6	32632558	32632574	Donor_Intron_5SS	1.1683	1.0107	0.1577	0.071	HLA-DQB1
T2D	rs9274514	6	32634243	32634275	Donor_Intron_5SS	1.2601	1.1527	0.1074	0.071	HLA-DQB1
T2D	rs28746846	6	32634261	32634275	Donor_Intron_5SS	1.5809	1.3917	0.1891	0.071	HLA-DQB1
T2D	rs72844399	6	32634264	32634275	Donor_Intron_5SS	1.354	1.0391	0.3149	0.071	HLA-DQB1

T2D	rs3213484	6	32725062	32725080	Acceptor_Exon_3SS	0.9443	0.6691	0.2751	0.217	HLA-DQB2
T2D	rs3213483	6	32726577	32726626	Donor_Intron_5SS	0.4287	0.5506	-0.1219	0.071	HLA-DQB2
T2D	rs16870880	6	32782018	32782096	Donor_Intron_5SS	-0.302	-0.2163	-0.0857	0.071	HLA-DOB
T2D	rs2071472	6	32784620	32784637	Donor_Intron_5SS	1.4924	1.6281	-0.1357	0.071	HLA-DOB
T2D	rs241442	6	32797168	32797176	Donor_Intron_5SS	1.2101	1.2905	-0.0803	0.071	TAP2
T2D	rs2288080	7	130357518	130357573	Donor_Intron_5SS	0.0161	0.0995	-0.0834	0.071	TSGA13
T2D	rs41291291	10	12191526	12191593	Acceptor_Intron_3SS	0.2216	0.4047	-0.1831	0.111	SEC61A2
T2D	rs1799859	11	17419279	17419343	Acceptor_Exon_3SS	1.4873	1.7237	-0.2364	0.217	ABCC8
T2D	rs4148634	11	17429872	17429938	Donor_Intron_5SS	1.6911	1.867	-0.1759	0.071	ABCC8
T2D	rs2190453	11	17533397	17533448	Donor_Intron_5SS	1.9982	2.0824	-0.0842	0.071	USH1C
T2D	rs2306615	11	72415329	72415378	Acceptor_Exon_3SS	0.3508	0.6262	-0.2753	0.217	ARAP1
T2D	rs861204	12	51237816	51237819	Donor_Exon_5SS	1.0261	1.2228	-0.1967	0.18	TMPRSS12
T2D	rs224454	12	51393116	51393127	Donor_Intron_5SS	0.047	0.1201	-0.0731	0.071	SLC11A2
T2D	rs224589	12	51399050	51399093	Donor_Intron_5SS	0.6914	0.7788	-0.0873	0.071	SLC11A2
T2D	rs427020	12	51404467	51404477	Donor_Intron_5SS	-0.1411	-0.0259	-0.1152	0.071	SLC11A2
T2D	rs7971308	12	51447506	51447560	Acceptor_Intron_3SS	0.2355	0.0967	0.1388	0.111	LETMD1
T2D	rs17525809	12	121592689	121592755	Donor_Exon_5SS	2.0742	2.3096	-0.2354	0.18	P2RX7
T2D	rs7132846	12	121614934	121614949	Acceptor_Ytract_3SS	1.2521	1.6617	-0.4096	0.367	P2RX7
T2D	rs12909056	15	91459475	91459485	Donor_Exon_5SS	0.3441	0.7432	-0.3991	0.18	MAN2A2
T2D	rs2001216	15	91498130	91498177	Donor_Exon_5SS	-0.8994	-0.6369	-0.2624	0.18	RCCD1
T2D	rs2290202	15	91512267	91512308	Donor_Intron_5SS	-0.3979	-0.2978	-0.1002	0.071	PRC1
T2D	rs2301826	15	91525197	91525210	Acceptor_Exon_3SS	0.7356	0.4523	0.2832	0.217	PRC1
T2D	rs12185460	18	6898415	6898434	Acceptor_BPS_3SS	-0.3565	-0.081	-0.2755	0.186	ARHGAP28

Supplementary Table 2: SplicePort score change thresholds by region.

Description	Interval[a]	2nd percentile[b]	M50[c]	M / n [d]
Exon 5' SS	GT 1-77;<br>donor -80 to -4	-0.343	-0.180	37 / 45
Core 5' SS	GT 78-80, 83-86;<br>donor -3 to -1, +3 to +6	-1.527	-1.104	73 / 75
Intron 5' SS	GT 87-162;<br>donor +7 to +82	-0.190	-0.071	11 /18
Intron 3' SS	AG 1-56;<br>acceptor -82 to -27	-0.231	-0.111	9 / 11
BPS 3' SS	AG 57-64;<br>acceptor -26 to -19	-0.448	-0.186	3 / 6
Ytract 3' SS	AG 65-79;<br>acceptor -18 to -4	-0.890	-0.367	29 / 30
Core 3' SS	AG 80, 83;<br>acceptor -3 or +1	-1.590	-1.455	17 / 18
Exon 3' SS	AG 84-162;<br>acceptor +2 to +80	-0.416	-0.217	48 / 56

[a]The first number (e.g. 1-77) is the position within window of 162 nucleotides (AG or GT at 81-82) used for SplicePort; the second number is relative to the splice site (standard nomenclature in which nucleotides upstream of the splice site are negative, those downstream are positive and there is no nucleotide 0).

[b]2nd percentile of all score changes due to single nucleotide substitutions in all human splice sites (mean within interval).

[c]M50 is the median of negative score changes found in *bona fide* mutations affecting splicing obtained by extensive surveys of the primary literature guided by text searches on PubMed and analysis of the Human Gene Mutation Database (Zaneta Franklin, unpublished; manuscript in preparation).

[d]Number of *bona fide* mutations used for our analysis. M is the number with negative score changes and n is the number of examples.

Supplementary Table 3: List of SNPs overlapping with direct splice sites.

#disease	chr	hg19_pos	snpid	Wild base	mutant base	strand	functional class	Gene (s)
BD	8	142170884	rs2289001	C	T	+	synsnp	DENND3
BD	14	21796784	rs3748361	G	C	+	missense	RPGRIP1
BD	18	166819	rs563155	T	C	+	synsnp	USP14
BD	19	35998362	rs4254439	A	C	-	synsnp	DMKN
CAD	6	31237162	rs1130863	C	G	-	missense	HLA-C
CAD	6	31237862	rs9264621	A	G	-	missense	HLA-C
CAD	6	31237987	rs41556321	G	A	-	missense	HLA-C
CAD	6	31238851	rs2308604	A	G	-	synsnp	HLA-C
CAD	6	31239378	rs1131123	A	C	-	missense	HLA-C
CAD	6	31239776	rs2074493	T	G	-	missense	HLA-C
CAD	6	31322303	rs2308655	G	C	-	missense	HLA-B
CAD	6	31323945	rs2596493	T	A	-	synsnp	HLA-B
CAD	6	31368540	rs2523452	G	C	+	UTR-5	MICA
CAD	6	31379134	rs1131897	C	G	+	missense	MICA
CAD	9	136268952	rs33919837	G	T	+	missense	C9orf96
CD	1	67773649	rs11209046	T	C	+	UTR-5	IL12RB2
CD	1	114447565	rs1217397	C	T	-	UTR-3	AP4B1
CD	1	155033308	rs11589479	G	A	+	synsnp	LOC100505666,ADAM15
CD	2	27730940	rs1260326	T	C	+	missense	GCKR
CD	4	48172266	rs2271173	C	T	-	synsnp	TEC
CD	6	31237162	rs1130863	C	G	-	missense	HLA-C
CD	6	31237862	rs9264621	A	G	-	missense	HLA-C
CD	6	31237987	rs41556321	G	A	-	missense	HLA-C
CD	6	31238851	rs2308604	A	G	-	synsnp	HLA-C
CD	6	31239378	rs1131123	A	C	-	missense	HLA-C
CD	6	31239776	rs2074493	T	G	-	missense	HLA-C

CD	6	31323945	rs2596493	T	A	-	synsnp	HLA-B
CD	6	31368540	rs2523452	G	C	+	intron,UTR-5	MICA
CD	9	139110654	rs12684650	G	A	-	synsnp	QSOX2
CD	9	139279173	rs10781510	C	T	-	synsnp	SNAPC4
CD	11	64004692	rs11558381	G	A	+	synsnp	VEGFB
CD	17	38064469	rs11078928	T	C	-	intron	GSDMB
CD	19	10449358	rs7258015	A	G	-	missense	ICAM3
CD	21	40178042	rs185407591	A	G	+	UTR-5,missense	ETS2
CD	21	40178043	rs190801053	G	C	+	UTR-5,missense	ETS2
CD	22	21846284	rs149272448	A	C	-	UTR-5	PI4KAP2
CD	22	22049783	rs12484060	C	T	+	synsnp	PPIL2
HT	11	100792335	rs75746929	G	A	+	synsnp	ARHGAP42
RA	6	29691303	rs2076183	G	A	+	synsnp	HLA-F
RA	6	29910801	rs1136692	C	A	+	missense	HLA-A
RA	6	29911319	rs41554520	G	A	+	synsnp	HLA-A
RA	6	29977145	rs356969	C	G	+	intron	HLA-J,ZNRD1-AS1
RA	6	32551948	rs17886882	C	A	+	missense	HLA-DRB1
RA	6	32811629	rs2071543	C	A	-	missense	PSMB8
RA	6	32862704	rs3749980	C	T	+	UTR-5	LOC100294145
RA	7	128578301	rs2004640	G	T	+	intron	IRF5
RA	8	11218893	rs2736277	A	G	+	intron	TDH
RA	17	38064469	rs11078928	T	C	-	intron	GSDMB
T1D	5	35700598	rs6451206	T	C	+	synsnp	SPEF2
T1D	6	32551886	rs17885959	G	A	-	missense	HLA-DRB1
T1D	6	32551948	rs17886882	C	A	+	missense	HLA-DRB1
T1D	12	9800926	rs7302081	C	T	+	UTR-5	LOC374443
T1D	12	9806405	rs35642909	G	A	+	intron	LOC374443
T1D	12	9833628	rs3764021	C	T	+	synsnp	CLEC2D

T1D	14	23830042	rs2231798	A	G	-	missense	EFS
T1D	17	7484101	rs25679	A	C	+	missense	CD68
T1D	17	7484101	rs9901673	C	A	+	missense	CD68
T1D	17	38064469	rs11078928	T	C	-	intron	GSDMB
T1D	19	10449358	rs7258015	A	G	-	missense	ICAM3
T2D	6	32521155	rs79811264	C	A	-	missense	HLA-DRB6
T2D	6	32548026	rs9269744	G	C	-	missense	HLA-DRB1
T2D	6	32551886	rs17885959	G	A	-	missense	HLA-DRB1
T2D	6	32551948	rs17886882	C	A	+	missense	HLA-DRB1
T2D	6	32811629	rs2071543	C	A	-	missense	PSMB8
T2D	8	95881041	rs114055881	G	A	+	intron	INTS8
T2D	10	12209752	rs6686	A	G	+	synsnp	SEC61A2,NUDT5
T2D	12	51203371	rs1129406	C	T	+	synsnp	ATF1
T2D	12	54911426	rs2270581	C	T	+	missense	NCKAP1L

Supplementary Table 4: Mechanisms for category A proteins

Disease	mechanism	Number of proteins	Protein names
BD	direct splice	1	USP14
BD	expression	1	CACNB3
CAD	expression	6	CELSR2,PSRC1,SORT1,GPR22,CDKN2B,LIPA
CAD	Low impact missense	2	AQP10,IL6R
CD	expression	6	ADAM15,MUC1,IL19,PTGER4,NOD2,UBE2L3
CD	High impact missense	1	MST1
CD	Low impact missense	1	NOD2
CD	Splice Port	2	ATG16L1,SF3A1
HT	direct splice	1	ARHGAP42
RA	expression	1	CD40
RA	High impact missense	1	PTPN22
T1D	expression	5	CLECL1,SH2B3,CTSH,SMARCE1,UBASH3A
T1D	High impact missense	1	PTPN22
T2D	expression	5	PPARG,CCNA2,TP53INP1,CDKN2B,NUCB2
T2D	Low impact missense	1	KCNJ11
T2D	Splice Port	1	WFS1

Supplementary Table 5. eQTL data for the 16 selected genome-wide eQTL association studies

Study ID	Samples (size)	Cell type	Genotyping	Phenotyping	eQTL associations	exSNPs	exGenes
HA	HapMap CEU (30)	LCL	HapMap Project	Illumina Human WG-6	3858	3686	239
	HapMap CHB (45)				4066	3780	253
	HapMap JPT (45)				5254	5061	274
	HapMap YRI (30)				3524	3283	306
BR	Caucasians (193)	Brain Cortex	Affymetrix 500K	Illumina HumanRefseq-8	624	545	209
AS	Childhood Asthma (206)	LCL	1. Illumina Human-1<br>2. Illumina HumanHap 300	Affymatrix HG-U133	21116	12121	2632
LV	Caucasian liver donors (427)	Liver cell	1. Illumina 650Y<br>2. Affymetrix 500K	Custom ink-jet microarray	4362	2527	3824
HA2	30 HapMap CEU (30)	LCL	HapMap Project	Affymetrix GeneChip	4453	3699	722
	30 HapMap YRI (30)			Human Exon 1.0	5027	4086	1659
3C	Caucasians (75)	LCL	Illumina 550K	Illumina Human WG-6	554	544	436
		Fibroblast			522	508	424
		T-cell			546	540	429
MO	German (1490)	Monocyte	Affymetrix 6.0	Illumina Human HT-12	37694	29948	2752
HRC	HapMap CEU (60)	LCL	HapMap Project	RNA-Seq	8908	3896	930
HRY	HapMap YRI (69)	LCL	HapMap Project	RNA-Seq	799	779	786
BR2	Caucasians (150)	Cerebellum	Illumina Infinium	Illumina HumanRef-8	5243	4399	317
		Frontal cortex	HumanHap 550		5512	5198	329
		Temporal			5335	4059	385

		cortex			3411	3284	275
		Pons					
SKN	Healthy skin individuals (57)	Skin	Perlegen Sciences array	Affymatrix HG-U133	5410	4782	222
LV2	Liver donors (266)	Liver cell	1. Illumina 610<br>2. Illumina HumanHap 550	1. Agilent-014850<br>2. Illumina HumanRef-8	1170	1161	1170
IM	British (288)	Monocyte	Illumina Human	Illumina HumanHT-12	33740	28956	6063
		B-cell	OmniExpress-12		22453	20333	5449
MuTHER	Caucasian female twins	LCL	Illumina	Illumina Human HT-12	211977	149684	3945
	(~160)	Skin	1. HumanHap 300		103537	82933	2495
		Adipose	2. HumanHap 610Q		138885	109689	3136
			3. 1M-Duo				
			4. 1.2MDuo 1M				
MRC	Childhood Asthma (MRCA: 405) & Atopic Dermatitis (MRCE: 950)	LCL	1. Illumina Human-1<br>2. Illumina HumanHap 300	1. Affymetrix HG-U133<br>2. Illumina Human WG-6	176848	109763	1251
E-GEUV	1000 Genome - EUR (373)	LCL	1000 Genome Project	Illumina HiSeq 2000	390813	281446	3048
	1000 Genome - YRI (89)	LCL			19314	16932	472
